# Innate Responses to the Former COVID-19 Vaccine Candidate CVnCoV and Their Relation to Reactogenicity and Adaptive Immunogenicity

**DOI:** 10.3390/vaccines12040388

**Published:** 2024-04-06

**Authors:** Olaf-Oliver Wolz, Dominik Vahrenhorst, Gianluca Quintini, Christina Lemberg, Sven D. Koch, Sarah-Katharina Kays, Lisa Walz, Neeraja Kulkarni, Michael Fehlings, Peter Wengenmayer, Jana Heß, Lidia Oostvogels, Sandra Lazzaro, Philipp von Eisenhart-Rothe, Philipp Mann

**Affiliations:** 1 CureVac SE, 72076 Tübingen, Germany; dominik.vahrenhorst@curevac.com (D.V.); gianluca.quintini@curevac.com (G.Q.); christina.lemberg@curevac.com (C.L.); sven.koch@curevac.com (S.D.K.); peter.wengenmayer@curevac.com (P.W.); cornelia.oostvogels@orange.fr (L.O.); sandra.lazzaro@curevac.com (S.L.); philipp.mann@curevac.com (P.M.); 2 ImmunoScape Pte Ltd., Singapore 139954, Singapore; neeraja.kulkarni@immunoscapre.com (N.K.); michael.fehlings@immunoscape.com (M.F.)

**Keywords:** COVID-19, SARS-CoV-2, immunogenicity, reactogenicity, mRNA vaccines, systems vaccinology

## Abstract

Vaccines are highly effective at preventing severe coronavirus disease (COVID-19). With mRNA vaccines, further research is needed to understand the association between immunogenicity and reactogenicity, which is defined as the physical manifestation of an inflammatory response to a vaccination. This study analyzed the immune response and reactogenicity in humans, post immunization, to the former SARS-CoV-2 mRNA investigational vaccine CVnCoV (CV-NCOV-001 and CV-NCOV-002 clinical trials). Immunogenicity was investigated using whole-blood RNA sequencing, serum cytokine levels, and SARS-CoV-2-specific antibodies. The T cell responses in peripheral blood were assessed using intracellular cytokine staining (ICS) and high-dimensional profiling in conjunction with SARS-CoV-2 antigen-specificity testing via mass cytometry. Reactogenicity was graded after participants’ first and second doses of CVnCoV using vaccine-related solicited adverse events (AEs). Finally, a Spearman correlation was performed between reactogenicity, humoral immunity, and serum cytokine levels to assess the relationship between reactogenicity and immunogenicity post CVnCoV vaccination. Our findings showed that the gene sets related to innate and inflammatory immune responses were upregulated one day post CVnCoV vaccination, while the gene sets related to adaptive immunity were upregulated predominantly one week after the second dose. The serum levels of IFNα, IFNγ, IP-10, CXCL11, IL-10, and MCP-1 increased transiently, peaking one day post vaccination. CD4^+^ T cells were induced in all vaccinated participants and low frequencies of CD8^+^ T cells were detected by ex vivo ICS. Using mass cytometry, SARS-CoV-2 spike-specific CD8^+^ T cells were induced and were characterized as having an activated effector memory phenotype. Overall, the results demonstrated a positive correlation between vaccine-induced systemic cytokines, reactogenicity, and adaptive immunity, highlighting the importance of the balance between the induction of innate immunity to achieve vaccine efficacy and ensuring low reactogenicity.

## 1. Introduction

Within 12 months of the World Health Organization (WHO) declaring that COVID-19 was a global pandemic, Comirnaty BNT162b2 (BioNTech/Pfizer) and Spikevax mRNA-1273 (Moderna) mRNA vaccines were approved for use around the world [1,2,3]. The mRNA technology platform has allowed for the rapid development and production of vaccines with the flexibility to be adjusted to new variants. Since the licensing of the original COVID-19 vaccines in 2020, several other vaccine candidates have been developed [4,5].

CVnCoV is a lipid nanoparticle (LNP)-encapsulated, chemically unmodified mRNA vaccine candidate based on CureVac’s mRNA vaccine platform and encoding a stabilized full-length ancestral SARS-CoV-2 spike protein. A phase I dose-escalation study (CV-NCOV-001) using 2–20 µg of CVnCoV demonstrated an acceptable safety profile in adults aged 18–60 years, and two vaccine doses, administered 28 days apart, induced neutralizing antibodies, in addition to binding IgG antibodies against both the SARS-CoV-2 spike protein and its receptor binding domain (RBD) [6]. Thereafter, a randomized phase IIa study (CV-NCOV-002) was conducted in adults aged 18–60 years and >60 years of age with dose levels of 6 and 12 µg of CVnCoV [7]. This study confirmed that two doses of 12 µg were safe and induced a robust humoral response, including immune memory responses. Consequently, the 12 µg CVnCoV dose was selected for a phase III clinical trial. In the phase IIb/III randomized, observer-blinded, placebo-controlled clinical trial (HERALD, CV-NCOV-004), its overall efficacy against symptomatic COVID-19 was 48.2% and its efficacy against moderate-to-severe COVID-19 was shown to be 70.7% [8].

The authorization of new vaccines depends on their immunogenicity, clinical efficacy, and safety. Reactogenicity refers to the symptoms occurring as a result of the inflammatory response to a vaccination [9], and the frequency and severity of reactogenicity is likely to affect vaccine compliance. Reactogenicity assessments for vaccine candidates typically include local signs and symptoms such as pain or swelling around the injection site, and systemic events including fever, fatigue, and headaches [9]. Immune cell activation must occur to trigger an effective vaccine response, although it is expected that post-vaccine inflammatory cytokine release and innate immune cell activation are associated with reactogenicity [10]. In addition to an antibody response, the induction of a T cell response mediated by CD4^+^ Th1 and cytotoxic CD8^+^ T cells is an important requirement of vaccine designs [11].

Investigations of the potential correlations between reactogenicity and innate and adaptive immunogenicity are required to gain a better understanding of their vaccine-induced mechanisms, with the eventual aim of reducing reactogenicity and increasing vaccine compliance whilst maintaining sufficient immunogenicity. The association between reactogenicity and immunogenicity has been explored for many vaccines, but, since mRNA technology is still in its infancy and current findings are inconsistent, more data are needed [12,13,14,15,16,17,18]. Investigations of the BNT162b2 mRNA vaccine suggest that its reported reactogenicity may not reflect antibody production [12,13,18], whilst other studies report that reactogenicity correlates with a stronger immune response. For example, a fever ≥ 38 °C was associated with higher spike-specific IgG antibody titers [16].

A prospective cohort study in healthcare workers (HCWs) demonstrated that the degree of local reaction after the first and second doses of the BNT162B2 vaccine was not associated with IgG titers and neutralizing antibodies, in an age- and sex-adjusted multivariate analysis [13]. Another study investigating responses to both the BNT162b2 and mRNA-1273 vaccines concluded that systemic symptoms remained associated with greater antibody responses in multivariable-adjusted models, including age and sex [17]. A further study investigating vaccination with BNT162b2 in HCWs indicated that systemic reactions after the first dose correlated with pre-existing cellular immunity, elicited by a prior SARS-CoV-2 infection or cross-reactivity, though reactogenicity following the second vaccine dose appeared to have an immunity-boosting effect [14]. These inconsistencies highlight the need to understand the biological mechanisms and characterize the immunological response induced by each investigational vaccine.

The humoral response, i.e., the binding and neutralizing of antibodies to CVnCoV, in phase I and phase II clinical trials (CV-NCOV-001 and CV-NCOV-002) has previously been published [6,7]. The purpose of this manuscript was to analyze the data collected from both trials to further investigate the interplay between CVnCoV-induced gene expression, humoral immunity, cytokine responses, reactogenicity, and T cell-mediated responses.

## 2. Materials and Methods

### 2.1. Trial Design of CV-NCOV-001 and CV-NCOV-002

CV-NCOV-001 (ClinicalTrials.gov identifier: NCT04449276) was a first-in-human, phase I, partially observer-blind, placebo-controlled, dose-escalation clinical trial conducted in healthy adults between 18 and 60 years of age. The trial evaluated the safety, reactogenicity, and immunogenicity of different CVnCoV dose levels ranging from 2 µg to 20 µg [6].

CV-NCOV-002 (ClinicalTrials.gov identifier: NCT04515147) was a phase II, partially observer-blind, active-controlled, dose-confirmation trial to assess the safety and immunogenicity of provisionally selected CVnCoV dose levels of 6 µg and 12 µg in adults >60 years and 18–60 years of age [7,19].

In both clinical trials, two intramuscular vaccinations of CVnCoV, at the same dose level, were administered 28 days apart, on Day 1 (baseline) and Day 29. The SARS-CoV-2 serostatus of participants was determined by testing for antibodies against its nucleocapsid (N) protein at each timepoint. If no antibodies against its N protein were detected, the participant was considered naïve and given seronegative status. If antibodies against N protein were detected, the participant was considered to have been pre-exposed to SARS-CoV-2 and was defined as seropositive.

For the CV-NCOV-002 trial, age-appropriate control vaccines included a licensed hepatitis A vaccine (Havrix, GSK, Rixensart, Belgium) recommended for use in the age group 18–60 years, and a licensed pneumococcal vaccine (Prevenar 13, Pfizer, Dublin, Ireland) recommended for use in the age group >60 years. Active control vaccines were administered according to the manufacturers’ instructions.

### 2.2. CVnCoV Reactogenicity Grading Used for Correlation Analysis

Reactogenicity in the CV-NCOV-001 and -002 clinical trials was graded after the first and second dose of CVnCoV, in terms of the vaccine-related solicited adverse events (AEs) per subject. The AEs, recorded across 12 local and systemic signs/symptoms, were graded according to their severity—0 (absent), 1 (mild), 2 (moderate), and 3 (severe)—using the FDA grading scale [20]. A subsequent correlation analysis was performed utilizing the maximum severity grade across all signs/symptoms.

### 2.3. RNA-Sequencing

Whole blood samples were collected from participants in the CV-NCOV-001 trial. In total, 210 samples were analyzed from 36 participants (12 participants each from the 2, 12, and 20 µg dose groups) for up to six timepoints (i.e., study visits: Day 1, Day 2, Day 8, Day 29, Day 30, and Day 36). Blood was collected in PAXgene blood RNA tubes (QIAGEN, Hilden, Germany) before mRNA isolation, library preparation, and sequencing at CeGaT AG (Tubingen, Germany). The depletion of globin and rRNA was performed during library construction. Next-generation sequencing (NGS) of the library was performed on a NovaSeq 6000 (Illumina, San Diego, CA, USA).

### 2.4. Gene Expression Profiling

Testing for significantly differentially expressed genes (DEGs), to compare either different timepoints to baseline (or Day 29) in one dose group (intra-dose-group comparisons) or values between different dose groups (inter-dose-group comparisons), was performed by CeGaT AG with DESeq2, as described in the Supplementary Methods. The DEGs from intra-dose-group comparisons underwent gene set enrichment analysis (GSEA) as previously described [21,22].

For the GSEA, the Blood Transcription Modules (BTMs) and HALLMARK Molecular Signatures Gene Set Databases were utilized [23,24]. Relevant BTM gene sets were divided into the following categories: Inflammatory, Interferon response, Monocytes, DC activation, Antigen presentation, Neutrophils, Platelets, NK cells, T cells, B cells, Plasma cells, and Cell cycle.

For each BTM or HALLMARK gene set, normalized enrichment scores (NESs) were calculated for the following timepoints: Days 2, 8, 29, 30, and 36. Different timepoints were then compared to Day 1 (baseline) or Day 29 in each CVnCoV dose group. The NESs are displayed as heatmaps; red/blue colors indicate significant (false discovery rate [FDR] < 0.01) upregulation/downregulation whilst the grey color indicates not enriched in GSEA or with non-significant FDR. Gene sets with at least one significantly up- or downregulated NES for the compared groups are presented. Further details are provided in the Supplementary Methods.

### 2.5. Serum Cytokine Analysis

Serum concentrations of the cytokines and chemokines listed in Table S1 were measured by Active Biomarkers (Lyon, France). For CV-NCOV-001, Day 1, 2, 8, 29, 30, and 36 samples from participants receiving 2–20 µg doses of CVnCoV were analyzed. For CV-NCOV-002, Day 1 and 2 serum samples from participants receiving 6 or 12 µg doses of CVnCoV or active control vaccines were analyzed. APRIL, BAFF, CCL4, CD40L, FasL, CXCL11, ICAM-1, IFN-β, IL-13, IL-17A, IL-5, IP-10, MCP-1, MCP-4, MDC, MICA, CXCL9, MIP-1α, SCF, and CD62L were assessed using the Luminex multiplex method (Bio-Plex^®^,Bio-Techne/R&D System; kit reference#LXSAHM-20). CXCL13 and IFNα were measured by a singleplex SIMOA HD-1 (Quanterix kits 102,635 and 100,860). The SIMOA SP-X Human 8-plex array kit (Quanterix 85-0321) was used to measure IFNγ, IL-1α, IL-6, and IL-10. Meso Scale Discovery (MSD) S-plex assays were used for the quantification of IL-2 (K151Z2S-1), IL-4 (K151A3S-1), IL-12(p70) (K151G3S-1), and TNF-α (K151E3S-1).

### 2.6. Immune Cell Analysis

#### 2.6.1. Peripheral Blood Mononuclear Cell Samples

Whole blood was obtained by venipuncture using sodium heparin-containing 8.0 mL BD Vacutainer^®^ CPT collection tubes on Day 1, 29, 36, and 211 for CV-NCOV-001 participants and Day 1, 29, and 43 for CV-NCOV-002 participants.

Peripheral blood mononuclear cells (PBMCs) were isolated within eight hours of blood sampling by density gradient centrifugation and cryopreserved in Cryo-SFM medium (PromoCell GmbH, Heidelberg, Germany) at 5 × 10 cells/mL/cryovial. Cryovials were transferred to a freezing container filled with room-temperature isopropanol and placed into a −80 °C freezer for at least 24 h. After 24–72 h, the PBMCs in cryovials were transferred to liquid nitrogen storage until further use.

Convalescent samples ranging from asymptomatic to moderate COVID-19 disease were obtained from CEVAC AG. Samples included one asymptomatic patient, four patients with mild disease and five patients with moderate disease. Ten pre-pandemic PBMC samples were also obtained from CEVAC AG for use as controls.

#### 2.6.2. T cell Intracellular Cytokine Staining

Intracellular cytokine staining (ICS) was used for the detection and characterization of SARS-CoV-2-spike-specific T cells in PBMCs [25].

Cells were stimulated, in the presence of anti-CD28 and anti-CD49d antibodies (BD Biosciences, Piscataway, NJ, USA), at 37 °C for 18 h with two custom pools of peptides (Sp1 and Sp2), consisting of 158 peptides covering the N-terminal residues 1–643 and the C-terminal residues 633–1272 of the SARS-CoV-2 spike protein, respectively (JPT Peptide Technologies, Berlin, Germany). As positive control, CEFX (JPT Peptide Technologies, Berlin, Germany) was used, while RPMI 1640 medium plus DMSO was used as the negative control. After two hours of stimulation, a protein transport inhibitor (GolgiPlug, BD Biosciences) was added. PBMCs were stained for the extracellular markers CD4 and CD8, fixed and permeabilized with Cytofix/Cytoperm (BD Biosciences), and stained for CD3, CD40L, IL-2, TNFα, and IFNγ (Table S2). Finally, cells were acquired on a Fortessa X-20 flow cytometer (BD Biosciences) and FlowJo version 9.9.6 software (FlowJo, Ashland, OR, USA). Results are presented as the frequency (%) of poly-functional cells in parent cell populations (CD3^+^CD4^+^ or CD3^+^CD8^+^ live lymphocytes). Poly-functional cells express at least two functional markers.

### 2.7. Mass Cytometry

A three-metal combinatorial peptide HLA Class I tetramer staining approach was used to screen for antigen-specific CD8^+^ T cells, as previously described [26,27]. Each sample was stained with a tetramer mixture containing 84 unique triple-coded peptide antigens (restricted to 6 HLAs [HLA-A*01:01, HLA-A*02:01, HLA-A*03:01, HLAA*11:01, HLA-A*24:02, and HLA-B*07:02]) derived from the SARS-CoV-2 spike protein and common virus control antigens, including Cytomegalovirus (CMV), Epstein–Barr virus (EBV), and Influenza (Flu). High-purity peptides were supplied by Genscript (Nanjing, China) or Mimotopes (Mulgrave, Australia). A full list of the peptides used for mass cytometry analysis is detailed in Table S3.

PBMCs were thawed at 37 °C, transferred into complete RPMI medium (10% heat-inactivated fetal calf serum [hiFCS], 1% penicillin/streptomycin/glutamine, 10 mM HEPES buffer, 55 µM 2-mercaptoethanol) supplemented with 50 units/mL Benzonase. Monocytes (CD14^+^) were depleted using column-based magnetic sorting (Miltenyi, Bergisch Gladbach, Germany). Cells from each participant were transferred into one well of a 96-well plate and washed once with cytometry buffer (PBS, 2% FCS, 2 nM EDTA, 0.05% sodium azide), and each sample (maximum 5 million cells/sample) was stained with 50 μL of tetramer cocktail for one hour at room temperature (RT). Cells were washed twice, and each sample was further stained for 30 min with 36 different metal-labelled antibodies (Table S4) and 200 µM cisplatin during the final five minutes of incubation to discriminate between live and dead cells.

Cells were washed and fixed in 2% paraformaldehyde (PFA) in Phosphate-Buffered Saline (PBS) for 30 min at RT. For intracellular staining, cells were incubated in 1X permeabilization buffer (BioLegend, San Diego, CA, USA) and incubated with metal-conjugated anti-Perforin for 30 min at RT. Cells were washed and longitudinal samples from the same individuals were barcoded with a unique combination of two distinct bromoacetamidobenzyl-EDTA-linked (Dojindo, Munich, Germany) metal barcodes (Pd-102, Pd-104, and PD108) for 20 min at RT. Cells were then washed and resuspended in a 250 nM iridium DNA intercalator (Standard BioTools Inc., San Francisco, CA, USA) in 2% PFA in PBS for 20 min at RT. Barcoded samples were pooled together and adjusted to 0.5 million cells/mL in H_2_O or CAS buffer (Standard BioTools Inc.), together with 1% equilibration beads for acquisition on a CyTOF^®^ Helios or XT mass cytometer (Standard BioTools Inc.).

After mass cytometry acquisition, samples were de-barcoded and gated on live CD3^+^ CD8^+^ T cells (CD45^+^ DNA^+^ cisplatin^-^ TCRγδ^-^ cells). Antigen-specific triple-metal-positive cells were identified using an automated peptide-MHC gating method [28] and bona fide antigen-specific T cells were further assessed for their frequencies and a phenotypic analysis. The percentages of antigen-specific T cells were calculated based on the percentage of the parent bulk CD8^+^ T cell populations. High-dimensional phenotypic profiles and sample distributions were illustrated using UMAPs [29].

### 2.8. Statistical Analysis

For the analysis of cytokine concentration fold changes shown in volcano plots, statistical testing was performed using Wilcoxon signed-rank test and Benjamini–Hochberg *p*-value adjustment for multiple testing using R software (Version 4.2.2) (Table S5). Cytokine concentrations ≤the lower limit of quantification (LLOQ) were set to LLOQ/2.

For the cytokine concentration analysis shown in bar graphs, statistical significance testing was performed using non-parametric rank-based methods, with the significance level defined as *p* ≤ 0.05. Herein, statistical significance testing was only performed for cytokines when 50% of the reported values were higher than the LLOQ. For comparisons of the Day 1 vs. Day 2 and Day 2 vs. Day 30 values in one dose group (paired testing of different timepoint values of individual participants), a two-tailed Wilcoxon signed-rank test was applied. To compare values of different dose groups at Day 2 and Day 30, Kruskal–Wallis ANOVA with Dunn’s multiple comparisons testing was performed against the lowest CVnCoV dose group (2 µg.) Data were analyzed using GraphPad Prism 9 software (GraphPad Software, La Jolla, CA, USA).

The frequencies of antigen-specific T cells assessed by mass cytometry were compared using Kruskal–Wallis tests with Dunn’s multiple comparisons testing. Statistical significance was defined as * *p* ≤ 0.05, ** *p* ≤ 0.01, *** *p* ≤ 0.001, or **** *p* ≤ 0.0001, and not significant (ns) if *p* > 0.05. Data analysis was performed using a custom Cytographer^®^, ImmunoScape’s cloud-based analytical software, FlowJo version 9.9.6 software (FlowJo, Ashland, OR, USA), and GraphPad Prism 9 software (GraphPad Software, La Jolla, CA, USA).

## 3. Results

### 3.1. Gene Expression Profiling after CVnCoV Vaccination

Total RNA sequencing and a whole-transcriptome analysis were performed from whole blood cells isolated from 36 CV-NCOV-001 trial participants immunized with 2 µg (low), 12 µg (mid), or 20 µg (high) doses of CVnCoV, on Day 1 and Day 29. The principal component analysis (PCA) results demonstrate that the Day 2 (one day post first dose) and Day 30 (one day post second dose) samples cluster separately from the other assessed timepoints, i.e., Days 1, 8, 29, and 36 (see PC1 on the *X* axis) (Figure S1). Separation by PC2 on *Y* axis is by the participants’ sex, with samples from females clustering in the upper part of the plot (Figure S1).

The analysis of significant DEGs suggests that, across all dose levels, transcriptional activity was highest one day post vaccination (Days 2 and 30), when compared with baseline (Day 1) or Day 29 (Table S6, and Figures S2 and S3). Furthermore, at one week post vaccination (Days 8 and 36), the number of DEGs had decreased, and by one month post first vaccination (Day 29) the DEGs had decreased further (Figure S2). The DEGs analyses across different dose groups suggested that, compared to the 2 µg dose, a greater number of DEGs were present on Days 2 and 30 for the higher CVnCoV doses (12 and 20 µg), but DEG numbers were similar when comparing the 12 µg against 20 µg dose group (Figure S3), indicating that differences in gene expression were less profound between mid and high CVnCoV dose ranges.

#### 3.1.1. Gene Transcription Activity Related to Innate and Inflammatory Immune Processes

**Figure 1:**
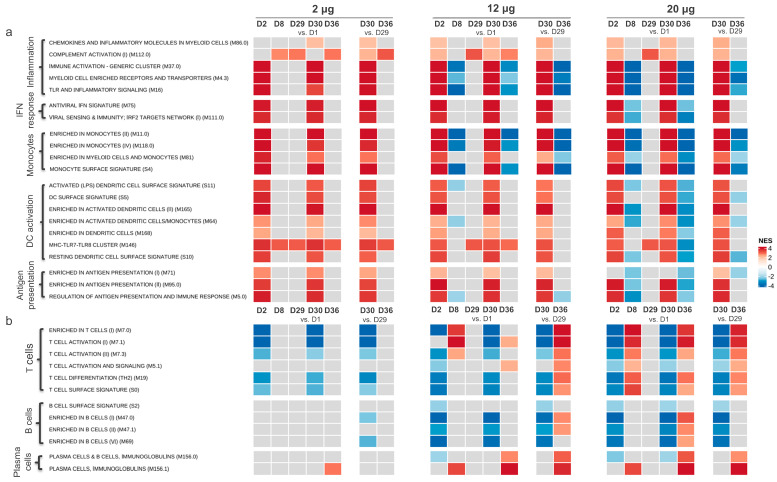
Adaptive and inflammatory immune response genes’ expression post CVnCoV vaccination. Gene set enrichment analysis (GSEA) from significantly differentially expressed genes (DEGs), summarized by the blood transcription modules (BTMs) of gene sets related to innate and inflammatory immune responses (**a**) and adaptive immune responses (**b**). Samples were from participants in the CV-NCOV-001 trial, vaccinated with 2, 12, or 20 µg CVnCoV (at least *n* = 11 participants per dose group who were SARS-CoV-2 seronegative at the shown timepoints). For participants who did not receive a second CVnCoV dose, their Day 30 and 36 timepoint values were excluded from analysis. Normalized enrichment score (NES) is in grey when not enriched in GSEA or with non-significant false discovery rate (FDR > 0.01); a colored NES indicates significance, with FDR < 0.01, red (positive score) indicates the upregulation and blue (negative score) the downregulation of listed gene sets between compared groups. D, Day; DC, dendritic cell; IFN, interferon, IRF2, interferon regulatory factor 2; LPS, lipopolysaccharides; MHC, major histocompatibility complex; n, number of participants; NFKB, nuclear factor kappa B; TNFA, tumor necrosis factor alpha; TLR, Toll-like receptor.

A GSEA of the DEGs was performed. The results were summarized as NESs between timepoints using the BTM (Figure 1) and HALLMARK gene set (Figure S4) databases. The analysis demonstrates that the gene expression related to innate immunity and inflammation was upregulated for all dose levels one day after the first and the second CVnCoV dose for most of the gene sets specifically relating to inflammation, IFN responses, monocytes, dendritic cell (DC) activation, and antigen presentation (Figure 1a). The upregulation of gene sets was transient, with expression returning to baseline levels within seven days of each CVnCoV dose (Day 8 and 36) for the 2 μg dose group. For the 12 μg and 20 μg dose groups, the expression of the gene sets relating to inflammation and monocytes decreased on Days 8 and 36, when compared to Day 1 expression levels.

The analysis of gene expressions using the HALLMARK database confirmed the upregulation of the gene sets relating to inflammation one day post first and second CVnCoV vaccinations across all vaccine dose groups (Figure S4).

#### 3.1.2. Gene Transcription Activity Related to Adaptive Immune Responses

The transcription of gene sets related to the adaptive immune response were also investigated post CVnCoV vaccination (Figure 1b). The results suggest that no upregulation of the T or B cell gene sets occurred in participants receiving the 2 µg dose of CVnCoV, at any timepoint. However, for the 12 µg dose group, the upregulation of gene sets relating to T cell activation and differentiation were detected by Day 8, with stronger upregulation occurring one week post second vaccination (on Day 36) compared to Day 29. The strongest upregulations of T cell-specific gene sets were detected in the 20 µg dose group on Day 8 and Day 36. Plasma cell BTMs were also upregulated for the 12 and 20 µg dose levels on Day 8 and 36, with the highest upregulation detected after the second CVnCoV dose.

### 3.2. Serum Cytokine Concentrations Post CVnCoV Vaccination

**Figure 2:**
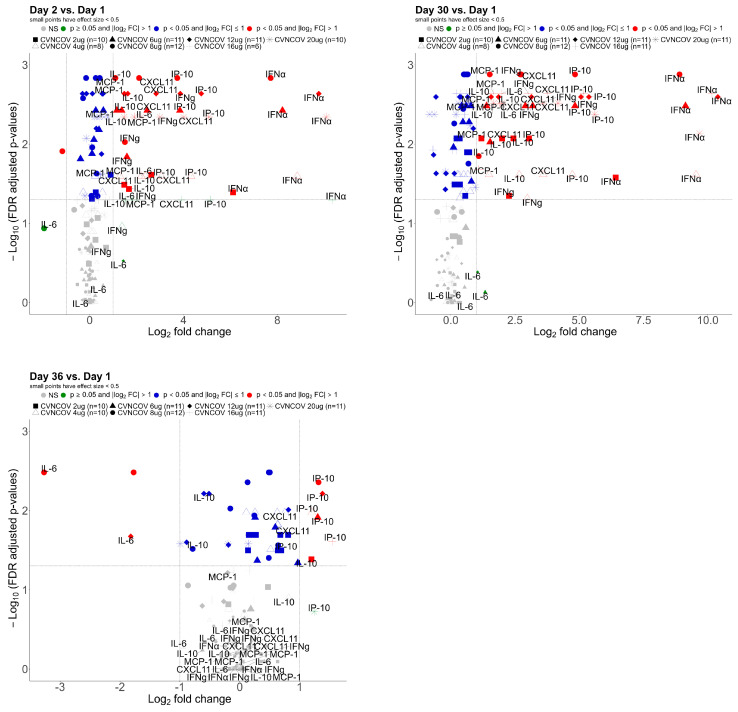
Serum cytokine fold changes post CVnCoV vaccination: Log2 fold changes in concentration of 29 cytokines in serum, after CVnCoV vaccination, on Day 2, 30, and 36, compared with Day 1. Data presented from 18–60-year-old CV-NCOV-001 adults who received two doses of 2–20 µg of CVnCoV and were SARS-CoV-2 seronegative at the shown time points. Cytokine concentrations ≤ lower limit of quantification (LLOQ) were set to LLOQ/2. The fold changes in red were <0.5 or >2 and the false discovery rate (FDR)-adjusted *p*-value < 0.05 (adjusted using the Benjmini–Hochberg method). CXCL, chemokine (C-X-C motif) ligand 1; IFNα, interferon alpha; IFNg, interferon gamma; IL, interleukin; IP-10, interferon-gamma inducible protein 10 kDa; MCP-1, membrane cofactor protein 1; n, number of participants; NS, not significant.

To further characterize the innate immune responses occurring after CVnCoV vaccinations, various serum cytokines and chemokines associated with inflammation (Table S1) were analyzed in the same cohort of CV-NCOV-001 participants (aged 18–60 years) whose samples were used for gene expression profiling. The fold changes in the concentrations of cytokines and chemokines from baseline are presented in Figure 2. The results demonstrate IFNα, IFNγ, IP-10, CXCL11, MCP-1, and IL-10 were the most influenced by CVnCoV, with up to 1000-fold increases in IFNα on Days 2 and 30.

**Figure 3:**
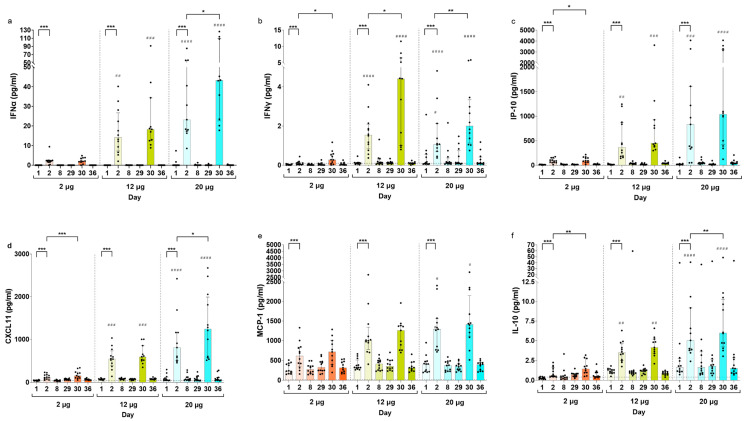
Serum cytokine concentration by dose and trial visit. IFNα (**a**), IFNy (**b**), IP-10 (**c**), CXCL11 (**d**), MCP-1 (**e**), and IL-10 (**f**) serum concentrations on Days 1, 2, 8, 29, 30, and 36 in SARS-CoV-2 seronegative participants vaccinated with two doses of 2, 12, or 20 µg of CVnCoV. If participants did not receive a second CVnCoV dose, their Day 30 and 36 values were excluded from analysis. Bars show median values with interquartile ranges. Dotted lines indicate the lower level of quantification (LLOQ) and values below LLOQ were reported as extrapolated values. Values below the detection limit were set to the lowest extrapolated value reported for the respective cytokine. Statistical comparisons in one dose group (indicated as *) were analyzed using a two-tailed Wilcoxon signed-rank test and comparisons of the Day 2 and Day 30 timepoints between dose groups were analyzed using a Kruskal–Wallis ANOVA with Dunn’s multiple comparisons testing, with 2 µg as the reference (indicated as #). Significance defined as */# *p* ≤ 0.05, **/## *p* ≤ 0.01, ***/### *p* ≤ 0.001, or #### *p* ≤ 0.0001. CXCL, chemokine (C-X-C motif) ligand 1; IFNα, interferon alpha; IFNy, interferon gamma; IL-10, interleukin 10; IP-10, interferon-gamma inducible protein 10 kDa; MCP-1, membrane cofactor protein 1.

Compared to baseline levels, the concentrations of IFNα, IFNγ, IP-10, CXCL11, MCP-1, and IL-10 cytokines were significantly increased on Day 2 across all CVnCoV doses (Figure 3, Figures S5 and S6). In addition, the levels of all listed cytokines were higher on Day 30 compared to Day 2, an increase that was most notable for concentrations of IFNγ and IFNα (Figure 3a,b). The results suggest that the levels of cytokines one day post vaccination generally increased with escalating dose concentrations of CVnCoV (Figure 3, Figures S5 and S6). This increase in the concentrations of the listed cytokines one day post vaccinations appears to be transient, as their levels returned to baseline within seven days of CVnCoV dose administrations.

The cytokine and chemokine concentrations were also determined for baseline (Day 1) and Day 2 (post CVnCoV) for participants in the CV-NCOV-002 trial (Figures S7 and S8). The analysis of cytokine induction confirmed that the serum concentrations of IFNα, IFNγ, IP-10, CXCL11, MCP-1, and IL-10 significantly increased on Day 2 compared to baseline in the SARS-CoV-2 seronegative participants of all ages receiving 6 and 12 µg doses (Figure S7). In the active control vaccine group, participants’ Day 2 cytokine concentrations were generally lower, and only their levels of IP-10 were significantly increased.

There were only a small number of SARS-CoV-2 seropositive participants in the CV-NCOV-002 study who received CVnCoV (one participant in the 6 µg dose group and three participants in the 12 µg dose group). However, for the 12 µg dose group, the Day 2 median values for IFNα, IP-10, CXCL11, and MCP-1 were higher in seropositive compared to seronegative participants, and significantly higher levels of IFNγ and IL-10 were found in seropositive participants (Figure S7).

To investigate whether participants’ ages affected their systemic secretion of these cytokines, the results were stratified into 18–60 years vs. over 60 years for participants from CV-NCOV-002 who received 12 µg of CVnCoV (Figure S8). The levels on Day 2 and the median fold-increases from Day 1 to Day 2 of IFNα, IFNγ, IP-10, CXCL11, MCP-1, and IL-10 were comparable between younger and older participants, with no statistically significant differences.

### 3.3. Correlation of CVnCoV Vaccination Reactogenicity, Humoral Immunity, and Serum Cytokine Levels

To investigate how innate immune response, humoral immunity, and reactogenicity post CVnCoV vaccination are linked, Spearman’s rank correlation coefficients were calculated. The pooled CV-NCOV-001 and CV-NCOV-002 data sets, including different dose and age groups, were used for this analysis to increase the sample size. For reactogenicity grade calculations after the first and second doses of CVnCoV, the maximum severity grade of all vaccine-related solicited AEs across all 12 local and systemic signs/symptoms was used [6,7]. For their correlation with humoral immunity, previously reported Day 43 peak titers of SARS-CoV-2 binding (spike- and RBD-specific IgG) and virus-neutralizing antibody titers (VNT), measured two weeks after the second dose, were used [6,7].

**Figure 4:**
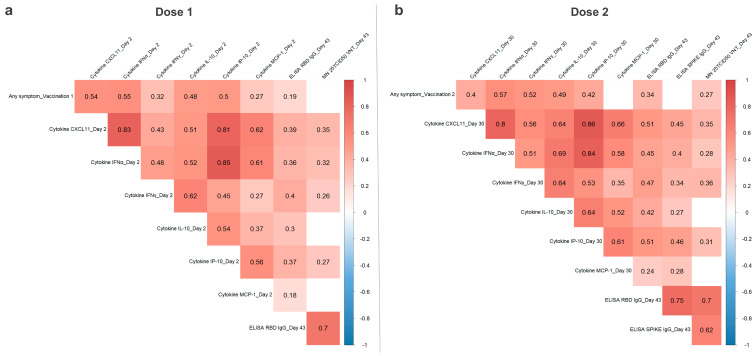
Correlation of reactogenicity, serum cytokine concentrations, and humoral responses after first and second doses of CVnCoV. Spearman correlation analysis from *n* = 131 pairs (pooled data from CV-NCOV-001 and -002 after first CVnCoV doses) (**a**) and *n* = 72 pairs (CV-NCOV-001 only), after their second dose, (**b**) of participants who received both CVnCoV doses (2, 4, 6, 8, 12, 16, or 20 µg) and who were SARS-CoV-2 seronegative for the shown time points. Fields with significant differences, *p* ≤ 0.05, are colored according to their Spearman correlation coefficient. Reactogenicity was calculated per participant as the maximum severity grade (0 = absent, 1 = mild, 2 = moderate, 3 = severe) of vaccine-related solicited adverse events (for any local or systemic sign/symptom) after both doses. Analysis was performed with VNT (virus neutralizing antibody titers), spike- or receptor-binding domain (RBD) IgG titers, and serum cytokine concentrations on the indicated days. CXCL, chemokine (C-X-C motif) ligand 1; ELISA, enzyme-linked immunosorbent assay; IgG, immunoglobulin G; IFNα, interferon alpha; IFNγ, interferon gamma; IL, interleukin; IP-10, interferon-gamma inducible protein 10 kDa; MCP-1, membrane cofactor protein 1; n, number of participants.

First-dose reactogenicity grades correlated moderately with the Day 2 concentrations of CXCL11, IFNγ, IFNα, IL-10, IP-10, and MCP-1, with the highest coefficients observed for IFNα and CXCL11 (Figure 4). Furthermore, a significant correlation between the concentrations of these cytokines demonstrates that participants with high CXCL11 levels were found to also have increased IFN-α concentrations in their serum (with a coefficient 0.83). There was also a moderate correlation between Day 2 cytokines and Day 43 RBD IgG and neutralizing antibody titers.

The pooled dose groups from CV-NCOV-001 participants were used for the correlation analysis after a second dose of CVnCoV (Figure 4). The results were similar to those observed after the first CVnCoV dose, with reactogenicity significantly correlating with cytokine levels (most prominently with coefficients of 0.57 and 0.52 for IFNα and IFNγ, respectively) on Day 30. There was a low to moderate correlation of Day 43 IgG titers and VNTs with both second-dose reactogenicity and Day 30 cytokine concentrations. Positive correlations between Day 2 and Day 30 cytokine concentrations were also observed (Figure S9). Finally, there were weak to moderate negative correlations observed between second-dose reactogenicity, Day 43 VNT and RBD IgG titers, and participants’ ages for both the 6 µg and 12 µg CVnCoV dose groups (Figure S10).

### 3.4. Cell-Mediated Immune Response Induced by CVnCoV

#### 3.4.1. Analysis of SARS-CoV-2 Spike-Specific T cell Responses using Flow Cytometry

**Figure 5:**
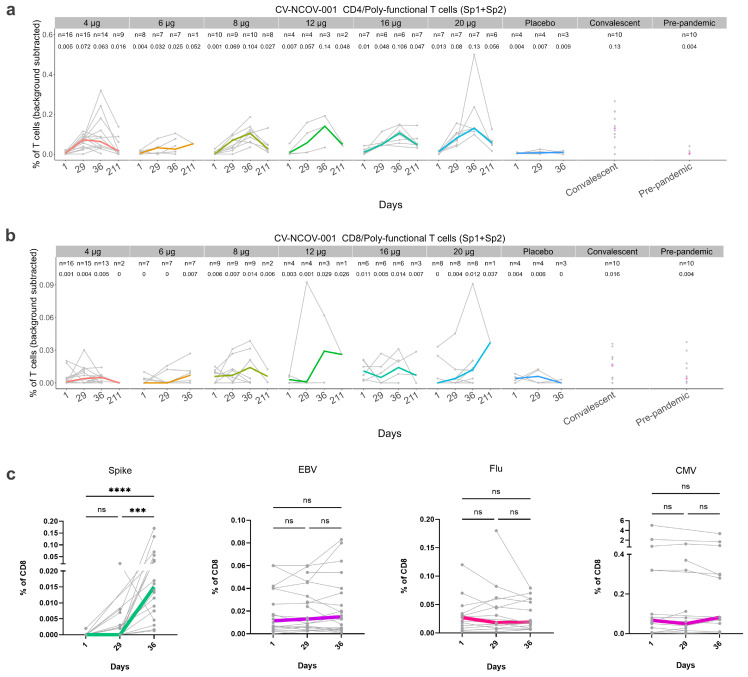
Frequencies of antigen-specific CD4+ and CD8+ T cells in longitudinal samples from CVnCoV-vaccinated trial participants. Using participant data from the CV-NCOV-001 clinical trial, samples from convalescent patients, and pre-pandemic samples, SARS-CoV-2-spike-specific CD4+ (**a**) and CD8+ T cells (**b**) were measured by intracellular cytokine staining after stimulation with two individual spike pools: spike pool 1 (Sp1) and spike pool 2 (Sp2). Sp1- and Sp2-induced T cell frequencies were background-subtracted and summed to a total frequency of poly-functional (PF) CD4+ and CD8+ T cells expressing at least two markers among IFNγ, TNFα, IL-2, and CD40L. For participants who showed a response on Day 29 and/or Day 36, their T cells were also measured on Day 211. The values above the data points indicate median of PF CD4+ and CD8+ T cells frequencies per time point. Virus-specific CD8^+^ T cell responses were also assessed by mass cytometry using pooled data from CV-NCOV-001 participants vaccinated with different dose levels of CVnCoV (**c**). Peptide HLA class I tetramer peptide antigens derived from CMV, EBV, influenza virus (Flu), or SARS-CoV-2 spike protein were used to detect antigen-specific CD8^+^ T cells. For (**a**–**c**), values from individual participants are represented by grey symbols connected by grey lines, whilst colored lines display group medians. Statistical analysis was performed using Kruskal–Wallis test followed by Dunn’s test for multiple comparisons. Statistical significance is defined as *** *p* ≤ 0.001, **** *p* ≤ 0.0001 or not significant (ns) if *p* > 0.05. CMV, cytomegalovirus; EBV, Epstein–Barr virus; Flu, influenza; PF, poly-functional; ns, not significant.

The effect of CVnCoV vaccination on the induction of T cells was investigated in both CV-NCOV-001 and CV-NCOV-002 trial participants. The T cells in PBMC samples were stimulated using two peptide pools (Sp1 and Sp2), which together span the entire amino acid sequence of the SARS-CoV-2 spike protein. After stimulation, antigen-specific poly-functional T cells were evaluated using a flow cytometry-based intracellular cytokine staining (ICS) assay and the results presented as the frequency (%) of either CD4^+^ or CD8^+^ T cells. The CD4^+^ and CD8^+^ T cell response from CV-NCOV-001 participants vaccinated with 4, 6, 8, 12, 16, and 20 µg doses of CVnCoV were analyzed on Day 1/baseline, 29, 36, and 211 (Figure 5a,b).

Spike-specific CD4^+^ T cells were induced after a single dose of CVnCoV (measured on Day 29) in most participants and further increased after their second dose (measured on Day 36). The induction of antigen-specific poly-functional CD4^+^ T cells was observed across all CVnCoV dose levels. Furthermore, the median levels of CD4^+^ T cells on Day 36 were comparable to those of COVID-19 convalescent samples (Figure 5a).

T cell persistency was assessed on Day 211 in participants shown to be responders on Day 29 and/or Day 36. T cell responders were defined as having a two-fold increase in their poly-functional CD4^+^ and/or CD8^+^ T cells compared to baseline levels or medium control. The analysis found that whilst the frequencies of CD4 ^+^ T cells were reduced on Day 211 compared to Day 36, for some participants they remained higher compared to baseline, placebo-controlled, and COVID-19 pre-pandemic samples (Figure 5a). Interestingly, the detection of spike-specific CD8^+^ T cells after the CVnCoV vaccination was considered to be low, with only a few participants showing increased CD8^+^ T cells on Day 29 or Day 36 compared to placebo or pre-pandemic controls. Nevertheless, the concentration of CD8^+^ T cells detected on Day 36, after vaccination with 8, 12, 16, or 20 µg CVnCoV, was comparable to that of the COVID-19 convalescent samples (Figure 5b).

Our recently published data on the cellular immune response to the 12 µg vaccine dose in the CV-NCOV-002 clinical trial [7] are further complemented in this study by the results from participants vaccinated with 6 µg of CVnCoV (Figure S11). The findings in this study confirm that the administration of 6 µg of CVnCoV induced spike-specific CD4^+^ T cells on Day 29 after the first dose’s administration, and there was a further increase in the response two weeks after the second dose (Day 43) [7]. As described for CV-NCOV-001, the frequencies of SARS-CoV-2-specific CD8^+^ T cells were low after the CVnCoV vaccinations, for both the 6 and 12 μg doses in the CV-NCOV-002 trial population [7] (Figure S11).

#### 3.4.2. Analysis of SARS-CoV-2 Spike-Specific CD8+ T Cells by Mass Cytometry

To identify and phenotypically profile the SARS-CoV-2 spike-specific CD8^+^ T cells in longitudinal PBMC samples from participants vaccinated with CVnCoV, we used a mass cytometry-based HLA class I tetramer staining strategy (Figure 5c and Figure S12). The frequencies of SARS-CoV-2 spike-specific CD8^+^ T cells ranged from 0.0013% to 0.17% of the total CD8+ T cells detected across all samples from CV-NCOV-001 (Figure 5c) and 0.0037% to 0.57% from CV-NCOV-002 (Figure S12).

For CV-NCOV-001, a total of 26 SARS-CoV-2 spike-specific CD8^+^ T cell populations were detected in 22 participants across all time points, with the majority of the T cells binding to YLQPRTFLL (HLA-A*02:01) and KCYGVSPTK (HLA-A*03:01) (Figure 5c, Table S7). In the CV-NCOV-002 trial, 19 SARS-CoV-2 spike-specific CD8^+^ T cell populations were detected across 24 participants, with QYIKWPWYI (HLA-A*24:02)-, NYNYLYRLF (HLA-A*24:02)-, and YLQPRTFLL (HLA-A*02:01)-specific CD8^+^ T cells being predominant across all samples (Figure S12, Table S7). In addition, several common viral antigen hits (CMV, EBV, influenza) were found across all individuals from the two cohorts. In both trials, a significant increase in spike-specific CD8^+^ T cell frequencies was observed over time, while no significant changes were found for common virus-specific CD8^+^ T cells (Figure 5c and Figure S12).

To assess the phenotypic profile of the SARS-CoV-2 spike-specific CD8^+^ T cells in comparison to the other CVnCoV-unrelated antigen-specific T cells detected across the same individuals, we combined the data from all time points and overlayed the phenotype of the T cells specific to each antigen category on two-dimensional UMAP plots, created based on the expression intensities of all phenotypic markers assessed (Figures S13 and S14). For CV-NCOV-001, SARS-CoV-2 spike-specific CD8^+^ T cells clustered together, characterized by their high expression of CD38, HLA-DR, PD-1, CD27, CD28, CD45RO, CD95, CXCR3, and CLA and low expression of CD39, CD45RA, CD71, CD57, and KLRG1 (Figure S13). The expression of these markers was also detected in samples from CV-NCOV-002, although the intensity of their expression was found to be lower than for the CV-NCOV-001 samples (Figure S14). There were some common markers detected across spike-specific CD8^+^ T cells and those specific to CMV, EBV, and influenza virus. These include CD45RO, CD95, CXCR3, and CD27, which are common on activated memory T cell phenotypes [30]. The additional markers detected on spike-specific T cells included CD38 and HLA-DR, which are considered to indicate immune activation [31].

## 4. Discussion

This study aimed to characterize the immune response to CVnCoV, a former mRNA COVID-19 vaccine candidate. The data collected from both phase I and II clinical trials were used to identify the correlations between CVnCoV-induced gene expression, cytokine and chemokine responses, reactogenicity, and adaptive immunity.

Gene expression profiling demonstrated that CVnCoV-induced gene transcription was related to the innate immune response one day post vaccination and that gene transcriptions present one week after vaccination were related to the adaptive immune response. The increased transcription of innate-immunity-related genes was also reported for the BNT162b2 mRNA vaccine, including the upregulation of interferon pathways one day after vaccine administrations [32]. However, in contrast to CVnCoV, where the gene sets relating to myeloid cells, monocytes, and activated DCs were already induced after the first dose, these BTMs were only activated one day post second vaccination with BNT162b2. Interestingly, several of the gene sets relating to inflammation appeared to be downregulated one week post vaccination (Days 8 and 36) in the 12 μg and 20 μg dose groups, an effect that appeared most pronounced for the gene sets with the highest upregulation one day post vaccination. In addition, we also observed the downregulation of gene sets relating to T cells, B cells, and plasma cells on Day 2 and Day 30 (one day post vaccination) which correlates to the reported drop in blood lymphocyte counts observed after administration for various mRNA vaccines [6,33,34]. Collectively, the observed downregulation of both the innate and adaptive gene sets at the described timepoints is thought to be related to the movement of lymphocytes from peripheral blood into secondary lymphoid tissue, where antigens are presented by innate cells to induce specific adaptive immunity, a process which is largely regulated by type 1 interferons [35].

Consistent with the innate-immunity-related transcription seen in GSEA, CVnCoV stimulated systemic cytokine and chemokine secretion. The serum levels of IFNα, IFNγ, IP-10, CXCL11, MCP-1, and IL-10, which are associated with inflammation and anti-viral processes, were, among other markers, most strongly increased in a dose-dependent manner one day after both the first and second vaccinations. IFNα, a strong modulator of the innate responses to viral infections, had the highest fold increase compared to the baseline levels after the first and second dose of CVnCoV. Compared to vaccination with BNT162b2 [32], the levels of IFNα were found to be up to 1000-fold higher after CVnCoV vaccination. Due to these effects being transient, with cytokine and chemokine concentrations returning to baseline levels one week after vaccination, CVnCoV is likely to stimulate systemic cytokine release via the RNA-sensing receptor pathways in innate immune cells [36].

The results demonstrated a trend of increased IFNγ, IFNα, CXCL11, IP-10, and IL-10 levels after the second administration of CVnCoV compared to after the first. This was most notable in the case of IFNγ, with more than two-fold higher levels for most dose groups on Day 30 compared to Day 2. Similarly, enhanced systemic cytokine responses, including IFNγ, were reported in mice immunized with BNT162b2, with Natural Killer (NK) cells and CD8^+^ T cells in the draining lymph nodes found to be the main sources of circulating IFNγ after the second dose’s administration [37]. In humans, serum IFNγ levels were found to be significantly increased in participants receiving the BNT162b2 vaccine one day after their second dose and correlated with spike-specific antibody levels [38]. This was also observed after the CVnCoV vaccination; however, IFNγ concentrations were approximately 20-fold higher after the BNT162b2 vaccine. The higher systemic IFNγ levels induced by the BNT162b2 vaccine may have contributed to its higher antibody-mediated protection against SARS-CoV-2, resulting in higher vaccine efficacy [1,2,3]. The reported reactogenicity after the BNT162b2 vaccination was higher after the second dose compared to the first [39]; however, the incidence of AEs (both local and systemic) were similar after the first and second doses of CVnCoV [7]. Since these two mRNA vaccines use similar LNP formulations, the observed differences in innate responses and reactogenicity are likely to be related to the distinct use of an unmodified (CVnCoV) compared to modified nucleobase N1-methylpseudouridine (BNT162b2) [40,41].

Both the induction of cytokines/chemokines and the reactogenicity symptoms after CVnCoV vaccination were transient, returning to baseline levels within a few days. As serum concentrations of IFNγ, IFNα, CXCL11, IP-10, and IL-10 positively correlated with the severity of symptoms, the observed higher cytokine levels measured on Day 30 compared to Day 2 could explain why a higher reactogenicity was reported after the second vaccine dose [8]. Further, the occurrence, duration, and fading of reported AEs correlated with the specific patterns of DEGs involved in the innate response after CVnCoV vaccination.

No differences in the induction of cytokines/chemokines on Day 2 (after the first CVnCoV dose) were observed when comparing trial participants in the 18–60-year-old and >60-year-old cohorts. However, the correlation analysis identified a negative correlation between age and reactogenicity, with lower reactogenicity reported in trial participants >60 years of age, suggesting that lower reactogenicity may not be due to the lower levels of cytokines/chemokines in this age group. In addition, negative correlations were also found between participants’ age and their titers of RBD-specific IgG and neutralizing antibodies, suggesting that lower humoral responses to CVnCoV were induced in older trial participants. In the CV-NCOV-002 trial specifically, the frequency of most solicited AEs (both local and systemic) were lower in participants over 60 years, regardless of whether it was their first or second vaccination or a dose level of 6 or 12 µg of CVnCoV was used [7]. Previous studies examining the effects of the BNT162b2 mRNA vaccination on the induction of cytokines/chemokines also concluded there was no differential effect on cytokine/chemokine profiles based on age. However, the age cut-off for this analysis was 50 years [38].

CVnCoV-induced CD4^+^ T cell responses specific to the SARS-CoV-2 spike antigen were comparable to those of COVID-19 convalescent patient samples [42,43,44,45]. However, circulating SARS-CoV-2-specific CD8^+^ T cells are less consistently observed after CVnCoV vaccination and in convalescent patients, which could, in part, be due to lymph nodes or injection sites being preferable locations for detecting CD8^+^ T cells, rather than peripheral blood [46]. Moreover, it has been shown that 90% of CD8^+^ T cell epitopes for SARS-CoV-2 are not derived from the spike protein [47]. Together, these findings could explain why poly-functional circulating CD8^+^ T cells with specificities to the spike protein are less frequently observed by flow cytometry-based ICS after a vaccination with CVnCoV. In addition, the 15-mer overlapping peptide pools spanning the spike protein’s primary sequence used for cell stimulation might not display optimal binding epitopes for the HLA class I molecules (usually 8- to 11-mers) recognized by specific CD8^+^ T cells [48,49,50].

By using a multiplexed peptide HLA class I tetramer-based CyTOF analysis, SARS-CoV-2 spike-specific CD8^+^ T cells were detected in 29 out of 46 (63%) SARS-CoV-2 naïve HLA-untyped participants after their vaccination with CVnCoV (CV-NCOV-001 and CV-NCOV-002). Due to the participants not being HLA-typed, it is likely that some participants may not have had an HLA type corresponding to the most prevalent HLA tetramers, hence the performed analysis might have underestimated the participant response rate of antigen-specific T cells against spike and control antigens.

The spike-specific CD8^+^ T cells induced by the CVnCoV immunization differed from the control-antigen-specific T cells and showed an CD45RO^+^CD45RA^-^CCR7^-^CD27^+^CD28^+^CXCR3^+^ early effector memory phenotype and early differentiation state, lacking late-differentiation markers such as CD57 and KLRG1. The co-expression of the activation markers CD38 and HLA-DR but the lack of early activation markers such as CD25 (IL-2Ra), which is required for proliferation during clonal expansion, or CD69, might indicate the peak of the T cell expansion phase. The T cell profiles observed in this study are similar to those observed in convalescent COVID-19 individuals [51]. Previous studies have highlighted that T cells confer protection against SARS-CoV-2, with virus-specific memory responses detected in individuals who have recovered from COVID-19, even in the absence of detectable antibodies [42]. Such activated effector memory CD8^+^ T cells (and SARS-CoV-2-specific T cells of other phenotypes), do not directly prevent infection as mediated by neutralizing antibodies, but potentially contribute to CVnCoV efficacy by protecting from severe COVID-19 disease [8].

After the HERALD phase III trial [8], CureVac decided to cease the development of CVnCoV and focus their efforts on the development of next-generation COVID-19 vaccine candidates. Considering this, investigation of immune responses and correlation to reactogenicity for future vaccine candidates can be compared with that of CVnCoV to better understand the differences between unmodified and modified mRNA in terms of reactogenicity and immunogenicity, as well as vaccine efficacy and compliance.

## 5. Conclusions

The results demonstrated that CVnCoV is biologically active across doses ranging from 2 to 20 µg and that it induced both innate and adaptive immune responses. The RNA sequencing results are in line with the serum cytokine responses related to innate immune activation and to adaptive immune responses, as demonstrated by antibody titers, and the T, B, and plasma cell activation detected one week after the administration of the second dose of CVnCoV. A detailed T cell analysis showed the induction of CD4^+^ Th1 T cells in all vaccinated participants and low frequencies of CD8^+^ T cells, as detected by ex vivo ICS. Despite the fact that we detected only a weak induction of spike-specific CD8^+^ T cells by ICS, peptide HLA class I tetramer staining using mass cytometry showed a clear induction of spike-specific CD8^+^ T cells with an activated effector memory phenotype.

As reactogenicity and early innate immune responses correlated positively, these factors should be considered for the development of future vaccine candidates.

## Data Availability

The data that support the findings of this study are included in the manuscript and/or are available upon request by qualified researchers from the corresponding author with an appropriate protocol for research use. The full data sets, including source data, are not publicly available due to them containing information that could compromise the research, participant privacy, or consent. Upon request for and approval of data transfer for re-analysis by qualified researchers, all personal information will be redacted to protect participant privacy.

## Supplementary Materials

The following supporting information can be downloaded at https://www.mdpi.com/article/10.3390/vaccines12040388/s1, Supplementary Methods: RNA-sequencing Analysis [21,22,23,24,52,53,54,55,56,57]; Table S1: Cytokines and Chemokines Included in Serum Cytokine Analysis; Table S2: Antibodies Used for ICS; Table S3: Peptides Used for Mass Cytometry Analysis; Table S4: Antibodies Used for Mass Cytometry; Table S5: R Packages Used for Analysis; Table S6: Differentially Expressed Genes Between Different Days and CVnCoV Dose Level Groups; Table S7: Frequencies of SARS-CoV-2 Spike-Specific CD8+ T Cells in Longitudinal Samples from CVnCoV-Vaccinated Participants from CV-NCOV-001 and CV-NCOV-002 Trials; Figure S1: Principal Component Analysis of Gene Expression Data. Principal component analysis (PCA) from expression data of genes with at least two sequencing reads; Figure S2: Significantly Differentially Expressed Genes Between Different Time Points per CVnCoV Dose Group; Figure S3: Significantly Differentially Expressed Genes on Different Days Between CVnCoV Dose Group; Figure S4: GSEA with HALLMARK Gene Sets After CVnCoV Vaccination; Figure S5: Serum Interferon Concentration by Dose and Trial Visit; Figure S6: Induction of Serum Cytokines Post CVnCoV Vaccination; Figure S7: Serum Cytokine Concentrations Post CVnCoV Vaccination By SARS-CoV-2 Serostatus; Figure S8: Serum Cytokine Concentrations by Trial Visit and Age Group; Figure S9: Correlation of Serum Cytokine Levels After First and Second Doses of CVnCoV Vaccine; Figure S10: Correlation of CVnCoV Reactogenicity and Humoral Immunity with Participant’s Age; Figure S11: Ex Vivo ICS Analysis of Spike-Specific CD4+ and CD8+ T Cell Responses Post Vaccination in CV-NCOV-002 clinical trial; Figure S12: Frequencies of Antigen-Specific CD8+ T cells in Longitudinal Samples from CVnCoV-Vaccinated Trial Participants; Figure S13: Phenotypes of Antigen-Specific CD8+ T cells From CVnCoV-Vaccinated Individuals; Figure S14: Phenotypes of Antigen-Specific CD8+ T cells From CVnCoV-Vaccinated Individuals.
