# Supplementary material for: Innate Responses to the Former COVID-19 Vaccine Candidate CVnCoV and Their Relation to Reactogenicity and Adaptive Immunogenicity
Source: Vaccines (Basel). 2024 Apr 6;12(4):388. doi: 10.3390/vaccines12040388 (PMC11053638; doi:10.3390/vaccines12040388)
Supplement: Supplementary file 1 [file vaccines-12-00388-s001.zip › vaccines-2910956-Supplementary-highlighted1.pdf]

Supplementary Methods

RNA-sequencing Analysis

Sample and library preparation: samples were divided into batches for RNA isolation and library preparation to avoid batch effects which could affect group comparisons. Whole blood was collected from study participants in PAXgene Blood RNA tubes (Qiagen, Hilden, Germany) and shipped to CeGaT for mRNA isolation, library preparation (KAPA RNA HyperPrep Kit with RiboErase y(HMR) Globin) and sequencing. Globin and rRNA were depleted in library construction. NGS library sequencing was on a NovaSeq 6000 (Illumina) with read length of 100 bp for read 1 and read 2 (in total 2x 100 bp). RNA sample quality was assessed by the RIN value and DV200 value using Qubit RNA (Thermo Fisher) and Fragment Analyzer (Agilent).

Transcriptome sequencing data analysis: demultiplexing of the sequencing reads was performed with Illumina bcl2fastq 2.20 and adapters were trimmed with Skewer 0.2.2. Reads with a Phred score of < 30 were removed using Trimmomatic 0.39. Trimmed raw reads were aligned to the human reference genome (hg19) with transcript annotation from Ensembl (GRCh37, v75)) using STAR 2.5.2b and multimappers removed. The quality of the FASTQ files were analyzed with FastQC. The read length after adapter trimming, the average base quality per read (as Phred score) and average GC content of the reads were examined via histograms.

Differential expression analysis: group comparisons for testing significant differentially expressed genes (DEGs) performed by CeGaT with DESeq2 [1] in R (version 3.6.1) [2]. DESeq2 uses a negative binomial generalized linear model to test for differential expression based on gene counts. As recommended by DESeq2, genes with less than two reads over all samples were removed and finally normalized. Sample RNA quality including possible sample outlier and sample characteristics were investigated by a PCA and dendrogram on rlog- transformed read counts. For each type of group comparison counts were modeled by the design formulas:

- A2, A3, A4: subject_ID + Day
- A5, A6, A7: Dose_group

Log2 fold changes were calculated, and the Wald test was applied for statistical testing with a significance level of 0.05 including a multiple testing correction by Benjamini-Hochberg [3] for adjusting the p-values. For each comparison, genes with a read count of zero in all compared samples or a read count of 1 in only one sample were excluded.

DEGs from group comparisons were further applied to gene set enrichment analysis (GSEA, Linux Version 4.1.0) [4,5] by CureVac Bioinformatics to the Blood Transcription Modules (BTM version 8 from October 2013 [6]) and HALLMARK molecular Signatures Database (MSigDB, version 7.4 [7]) gene set databases. The 50 HALLMARKs show a wide range of biological processes and were summarized from multiple founder gene sets to reduce variation and redundancy. The BTMs were identified from systems signatures from five human vaccines from a large-scale network integration of public human blood transcriptomes, and systems-scale databases in specific biological contexts. The 382 BTM sets (modules) are annotated by their function and focus more on processes related to immunity.

GSEA parameter: genes for GSEA were pre-ranked (according to metric described in Reimand et al. [8]). Firstly, all significant (cut-off adjusted P value <0.05) differentially expressed genes for each group comparison were extracted. A score per gene $s_{\text{gene } i}$ was calculated and finally all genes per group comparison were sorted in a decreasing order. The scoring metric uses the sign (*sign*) of the log2 fold change of each gene ($\text{log2FC}_{\text{gene } i}$) with negative logarithm (*log10)* of adjusted P value ($\text{padj}_{\text{gene } i}$) based on multiple testing using the Benjamini-Hochberg method:

$$s_{\text{gene } i} = sign(\text{log2FC}_{\text{gene } i})*(-log10(\text{padj}_{\text{gene } i}))$$

The ranked gene list per group comparison is received where top genes on the list represent up-regulation and genes at the bottom of the list down-regulation.

GSEA default settings were used, number of permutations was set to 10,000 and type of permutation was “genotype” because of pre-ranked gene list. To include HGNC (HUGO Gene Nomenclature Committee) gene annotations in the analysis report the command “collapse” was used and the chip annotation file Human_Gene_Symbol_with_Remapping_MSigDB.v7.4.chip.

Results from GSEA were visualized in R (4.0.4) as heatmaps using the ComplexHeatmap (2.6.2) package [9] and significant DEGs as bar plots with ggplot2 in R (4.0.4) [10].

Sensitivity: Official Use

Supplementary Tables

Table S1. Cytokines and Chemokines Included in Serum Cytokine Analysis

Cytokine Abbreviation	Abbreviation Meaning
**APRIL**	A-proliferation-inducing ligand
**BAFF**	B-cell activating factor
**CCL4**	C-C chemokine ligand 4
**CXCL9**	C-X-C chemokine ligand 9
**CXCL11**	C-X-C chemokine ligand 11
**CXCL13**	C-X-C chemokine ligand 13
**CD40L**	CD40 Ligand
**CD62L**	CD62 Ligand
**FasL**	Fas Ligand
**ICAM-1**	Intercellular adhesion molecule 1
**IFNα**	Interferon α
**IFNβ**	Interferon β
**IFNγ**	Interferon γ
**IL-1α**	Interleukin 1a
**IL-2**	Interleukin 2
**IL-4**	Interleukin 4
**IL-5**	Interleukin 5
**IL-6**	Interleukin 6
**IL-10**	Interleukin 10
**IL-12(p70)**	Interleukin 12(p70)
**IL-13**	Interleukin 13
**IL-17A**	Interleukin 17A
**IP-10**	Interferon gamma-induced protein 10
**MCP-1**	Monocyte chemotactic protein 1
**MCP-4**	Monocyte chemotactic protein 4
**MDC**	Macrophage-derived chemokine
**MICA**	MHC class I polypeptide-related sequence A
**MIP-1α**	Macrophage inflammatory protein 1α
**SCF**	Stem cell factor
**TNFα**	Tumour necrosis factor α

Sensitivity: Official Use

Table S2. Antibodies Used for ICS

Marker	Clone	Supplier	Catalogue Number
**Live/dead**	-	Invitrogen	L34966
**CD8**	SK1	BD Biosciences	641400
**CD4**	SK3	BD Biosciences	345770
**CD3**	UCHT1	BD Biosciences	557943
**CD40L**	TRAP1	BD Biosciences	555700
**IL-2**	MQ1-17H12	BD Biosciences	554567
**TNFα**	MAb11	BD Biosciences	557647
**IFNγ**	4S.B3	BD Biosciences	554551

Sensitivity: Official Use

Table S3. Peptides Used for Mass Cytometry Analysis

No.	Peptide name	Peptide sequence	Antigen	HLA
**1**	A*02:01_1	KLPDDFTGCV	S	A*02:01
**2**	A*02:01_2	LITGRLQSL	S	
**3**	A*02:01_3	RLNEVAKNL	S	
**4**	A*02:01_4	YLQPRTFLL	S	
**5**	A*02:01_5	RLQSLQTYV	S	
**6**	A*02:01_6	VLNDILSRL	S	
**7**	A*02:01_7	KIADYNYKL	S	
**8**	A*02:01_8	SIIAYTMSL	S	
**9**	A*02:01_9	LLFNKVTLA	S	
**10**	A*02:01_10	RLDKVEAEV	S	
**11**	A*02:01_11	HLMSFPQSA	S	
**12**	A*02:01_12	FIAGLIAIV	S	
**13**	A*02:01_13	ALNTLVKQL	S	
**14**	A*02:01_14	NLNESLIDL	S	
**15**	A*02:01_15	VVFLHVTYV	S	
**16**	A*02:01_C1	VLEETSVML	CMV-IE1	
**17**	A*02:01_C2	NLVPMVATV	CMV-pp65	
**18**	A*02:01_C3	CLGGLLTMV	EBV-LMP2A	
**19**	A*02:01_C4	FLYALALLL	EBV-LMP2A	
**20**	A*02:01_C5	GILGFVFTL	Flu-M1	
**21**	A*11:01_1	GVYFASTEK	S	A*11:01
**22**	A*11:01_2	TLKSFTVEK	S	
**23**	A*11:01_3	RLFRKSNLK	S	
**24**	A*11:01_4	NSASFSTFK	S	
**25**	A*11:01_5	VTYVPAQEK	S	
**26**	A*11:01_6	SSTASALGK	S	
**27**	A*11:01_7	ASANLAATK	S	
**28**	A*11:01_8	GTHWFVTQR	S	
**29**	A*11:01_9	MTSCCSCLK	S	
**30**	A*11:01_C1	ATVQGQNLK	CMV-pp65	
**31**	A*11:01_C2	AVFDRKSDAK	EBV(EBNA 3B)	
**32**	A*11:01_C3	SSCSSCPLSK	EBV(LMP2)	
**33**	A*11:01_C4	SIIPSGPLK	Flu-MP1	
**34**	A*24:02_1	NYNYLYRLF	S	A*24:02
**35**	A*24:02_2	VYSTGSNVF	S	
**36**	A*24:02_3	EYVSQPFLM	S	
**37**	A*24:02_4	QYIKWPWYI	S	
**38**	A*24:02_5	LYNSASFSTF	S	
**39**	A*24:02_6	PYRVVVLSF	S	
**40**	A*24:02_7	YYVGYLQPRTF	S	
**41**	A*24:02_8	VYSSANNCTF	S	
**42**	A*24:02_9	IYKTPPIKDF	S	
**43**	A*24:02_10	RFDNPVLPF	S	

Sensitivity: Official Use

No.	Peptide name	Peptide sequence	Antigen	HLA
**44**	A*24:02_11	PFFSNVTWF	S	
**45**	A*24:02_12	RFPNITNLCPF	S	
**46**	A*24:02_13	TYVPAQEKNF	S	
**47**	A*24:02_C1	QYDPVAALF	CMV-2 pp65 341-349	
**48**	A*24:02_C2	AYAQKIFKIL	CMV-5-IE1	
**49**	A*24:02_C3	RYSIFFDYM	EBV-10-EBNA3A NP	
**50**	A*24:02_C4	RYGFVANF	Flu-8-Pol	
**51**	A*24:02_C5	TYQWIIRNW	Flu-PB2	
**52**	A*03:01_1	GVYFASTEK	S	A*03:01
**53**	A*03:01_2	TLKSFTVEK	S	
**54**	A*03:01_3	RLFRKSNLK	S	
**55**	A*03:01_4	VTYVPAQEK	S	
**56**	A*03:01_5	QIYKTPPIK	S	
**57**	A*03:01_6	ASANLAATK	S	
**58**	A*03:01_7	MTSCCSCLK	S	
**59**	A*03:01_8	KVFRSSVLH	S	
**60**	A*03:01_9	RQIAPGQTGK	S	
**61**	A*03:01_10	KCYGVSPTK	S	
**62**	A*03:01_C1	KLGGALQAK	CMV (IE-1)	
**63**	A*03:01_C2	RLRAEAQVK	EBV (EMNA 3A)	
**64**	A*03:01_C3	ILRGSVAHK	Influenza (PR8)	
**65**	A*01:01_1	WTAGAAAYY	S	A*01:01
**66**	A*01:01_2	LADAGFIKQY	S	
**67**	A*01:01_3	LTDEMIAQY	S	
**68**	A*01:01_4	TSNQVAVLY	S	
**69**	A*01:01_C1	VTEHDTLLY	CMV (UL44)	
**70**	A*01:01_C2	YSEHPTFTSQY	CMV (pp65)	
**71**	A*01:01_C3	CTELKLSDY	Influenza (NP)-A01	
**72**	A*01:01_C4	VSDGGPNLY	Influenza (PB1)-A01	
**73**	B*07:02_1	SEPVLKGVKL	S	B*07:02
**74**	B*07:02_2	LPQGFSAL	S	
**75**	B*07:02_3	KPFERDISTEI	S	
**76**	B*07:02_4	QPYRVVVL	S	
**77**	B*07:02_5	LPFNDGVYF	S	
**78**	B*07:02_6	SPRRARSVA	S	
**79**	B*07:02_7	IPTNFTISV	S	
**80**	B*07:02_8	APHGVVFL	S	
**81**	B*07:02_C1	RPHERNGFTVL	CMV (pp65 #1)	
**82**	B*07:02_C2	RPPIFIRRL	EBV (EBNA 3A)	
**83**	B*07:02_C3	SPIVPSFDM	Influenza (NP)	
**84**	B*07:02_C4	QPEWFRNVL	Influenza (PB1)	

Sensitivity: Official Use

Table S4. Antibodies Used for Mass Cytometry

Marker	Clone	Catalogue number	Company
**CD45**	Y-89	HI30 3089003B	Standard BioTools Inc
**CD19**	HIB19	bl302202	BioLegend
**CD8**	SK1	bl344727	BioLegend
**CD14**	TUK4	MCA1568GA	BioRAD
**CD4**	SK3	bl344602	BioLegend
**CD57**	HNK-1	bl359602	BioLegend
**CD3**	UCHT1	bl300414	BioLegend
**TCRγ/δ1**	B1	331210	BioLegend
**PE2**	PE001	bl408102	BioLegend
**CD56**	NCAM16.2	bd559043	BD
**HLA-DR**	L243	bl307602	BioLegend
**CD27**	LG.7F9	14-0271-85	Invitrogen
**CD24**	ML5	311127	Biolegend
**CXCR5**	RF8B2	bd552032	BD
**CRTH2**	BM16	13294982	eBioscience
**CD95**	DX2	305602	BioLegend
**CD45RO**	UCHL1	304202	BioLegend
**CCR6**	G034E3	bl353402	BioLegend
**CD103**	B-Ly7	14-1038-82	eBioscience
**CD69**	FN50	bl310902	BioLegend
**KLRG1**	13F12F2	ebio16-9488-85	eBioscience
**CLA**	HECA-452	bl321302	BioLegend
**CXCR3**	49801	MAB160-100	R&D
**CD45RA**	H100	bd555486	BD
**PD-1**	J105	14-2799-80	invitrogen
**CD161**	HP-3G10	bl339902	BioLegend
**CD127**	A019D5	bl351302	BioLegend
**CD39**	A1	bl328202	BioLegend
**CCR5**	RF8B2	566191	Genetex
**CCR7**	150503	MAB197	R&D
**CD25**	M-A251	bl356102	BioLegend
**CD28**	CD28.2	bl302914	BioLegend
**CD71**	CY1G4	bl334102	BioLegend
**CD38**	HIT2	bl303502	BioLegend
**CCR4**	L291H4	bl359402	BioLegend
**Perforin**	B-D48	ab47225	Abcam
**CD16**	T24/31	BE0212	Standard BioTools Inc
**DNA**	Ir-191/193	DNA	Standard BioTools Inc
**CisPlatin live/dead**	Pt-195	CisPlatin live/dead	Sigma

[1]anti-TCRγδ is the PE-labelled primary antibody. [2] secondary antibody: anti-PE.

Sensitivity: Official Use

Table S5. R Packages Used for Analysis

Analysis	R Packages used
**CMI**	R 4.3.0
	renv 1.0.0
	tidyverse 2.0.0
	DT 0.28
	plyr 1.8.8
	dplyr 1.1.2
	plotly 4.10.2
	openxlsx 4.2.5.2
	readxl 1.4.3
	viridis 0.6.4
	scales 1.2.1
	data.table 1.14.8
	patchwork 1.1.2
	formattable 0.2.1
	EnvStats 2.8.0
	ggforce 0.4.1
	ggh4x 0.2.5
	gghighlight 0.4.0
	ggridges 0.5.4
	ggbeeswarm 0.7.2
	ggthemes 4.2.4
	gridExtra 2.3
	testthat 3.1.10
	shiny 1.7.4.1
	shinythemes 1.2.0
	shinyWidgets 0.7.6
	plotlywrappers 0.1.0
**Correlation analysis**	R 4.0.4
	shiny 1.7.1
	tidyverse 1.3.1
	DT 0.19
	plotly 4.9.4.1
	scales 1.1.1
	EnvStats 2.4.0
	heatmaply 1.3.0
	reshape2 1.4.4
	readxl 1.3.1
	ggridges 0.5.3
	viridis 0.6.1
	ggthemes 4.2.4
	corrplot 0.92
	xlsx 0.6.5
	dplyr 1.0.7
**Cytokine Volcano Plots**	R version 4.2.2
	stringr 1.5.0
	tidyverse 1.3.2
	EnhancedVolcano 1.16.0
	yaml 2.3.7

Sensitivity: Official Use

Analysis	R Packages used
	readxl 1.4.1
	dplyr 1.1.0
	rstatix 0.7.2
	openxlsx 4.2.5.1
	renv 0.17.0

CMI, cell mediated immunity.

Sensitivity: Official Use

Table S6. Differentially Expressed Genes Between Different Days and CVnCoV Dose Level Groups

Dose group A	Dose group B	Analysis	Day X	Day Y	Numbers of differentially expressed genes
**2 µg**	2 µg	A2	D29	D1	48
**2 µg**	2 µg	A2	D2	D1	9586
**2 µg**	2 µg	A2	D30	D1	8985
**2 µg**	2 µg	A2	D30	D29	8877
**2 µg**	2 µg	A2	D36	D1	58
**2 µg**	2 µg	A2	D36	D29	234
**2 µg**	2 µg	A2	D8	D1	53
**12 µg**	12 µg	A3	D29	D1	51
**12 µg**	12 µg	A3	D2	D1	16250
**12 µg**	12 µg	A3	D30	D1	16100
**12 µg**	12 µg	A3	D30	D29	15668
**12 µg**	12 µg	A3	D36	D1	207
**12 µg**	12 µg	A3	D36	D29	534
**12 µg**	12 µg	A3	D8	D1	1879
**20 µg**	20 µg	A4	D29	D1	58
**20 µg**	20 µg	A4	D2	D1	14355
**20 µg**	20 µg	A4	D30	D1	14501
**20 µg**	20 µg	A4	D30	D29	15442
**20 µg**	20 µg	A4	D36	D1	868
**20 µg**	20 µg	A4	D36	D29	833
**20 µg**	20 µg	A4	D8	D1	559
**2 µg**	12 µg	A5	D1	D1	6
**2 µg**	12 µg	A5	D2	D2	3132
**2 µg**	12 µg	A5	D29	D29	0
**2 µg**	12 µg	A5	D30	D30	3677
**2 µg**	12 µg	A5	D36	D36	0
**2 µg**	12 µg	A5	D8	D8	501
**2 µg**	20 µg	A6	D1	D1	3
**2 µg**	20 µg	A6	D2	D2	6433
**2 µg**	20 µg	A6	D29	D29	5
**2 µg**	20 µg	A6	D30	D30	6587
**2 µg**	20 µg	A6	D36	D36	10
**2 µg**	20 µg	A6	D8	D8	1
**12 µg**	20 µg	A7	D1	D1	8
**12 µg**	20 µg	A7	D2	D2	0
**12 µg**	20 µg	A7	D29	D29	1
**12 µg**	20 µg	A7	D30	D30	2
**12 µg**	20 µg	A7	D36	D36	1
**12 µg**	20 µg	A7	D8	D8	0

Numbers of significantly differentially expressed genes (DEGs) between different Days (D) X and Y or CVnCoV dose groups A and B. Significant with adjusted p-value <0.05. Analysis from at least n=11 SARS-CoV-2 seronegative participants per dose group and timepoint. For participants who did not receive a second CVnCoV dose, Day 30 and 36 timepoint values were excluded from analysis.
n, number of participants

Sensitivity: Official Use

Table S7. Frequencies of SARS-CoV-2 Spike-Specific CD8+ T Cells in Longitudinal Samples from CVnCoV Vaccinated Participants from CV-NCOV-001 and CV-NCOV-002 Trials

**CV-NCOV-001**	**Day 1**	**Day 29**	**Day 36**
Participant samples tested (n)	20	18	20
Participant samples with hits (n)	1	7	15
Number of hits	1	7	18
Participants with different hits on the same day (n)	0	0	3
**CV-NCOV-002**	**Day 1**	**Day 29**	**Day 43**
Participant samples tested (n)	24	21	21
Participant samples with hits (n)	0	10	9
Number of hits	0	10	9
Participants with different hits on the same day (n)	0	0	0

A hit is defined as the detection of a SARS-CoV-2 spike derived tetramer-binding $CD8^+$ T cell.
For CV-NCOV-001, a total of 22 participants were included in the analysis, but 2 baseline samples were not available. For 1 participant who was tested seronegative for SARS-CoV-2, there was a hit detected on baseline. For CV-NCOV-002, a total of 24 participants were included in the analysis.

Sensitivity: Official Use

Supplementary Data

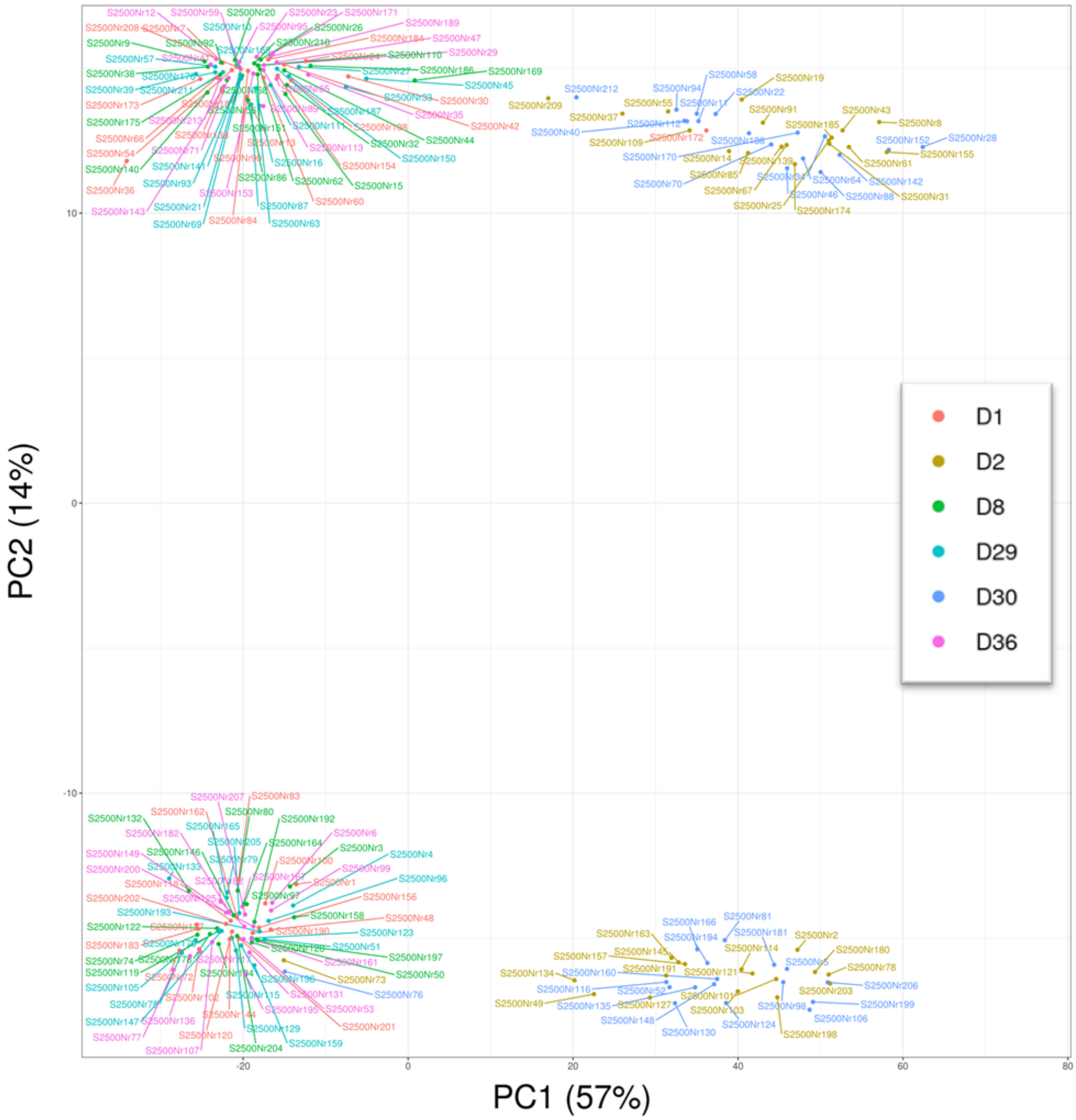

Figure S1. Principal Component Analysis of Gene Expression Data. Principal component analysis (PCA) from expression data of genes with at least two sequencing reads. The percentage values on the axes describe how much of the variance between samples is captured in this principal component (PC). Samples (S) (n=210) are colored according to Days (D) group and are from SARS-CoV-2 seronegative participants vaccinated with 2, 12 or 20 µg doses of CVnCoV. For participants who did not receive a second CVnCoV dose, Day 30 and 36 timepoint values were excluded from analysis.

Sensitivity: Official Use

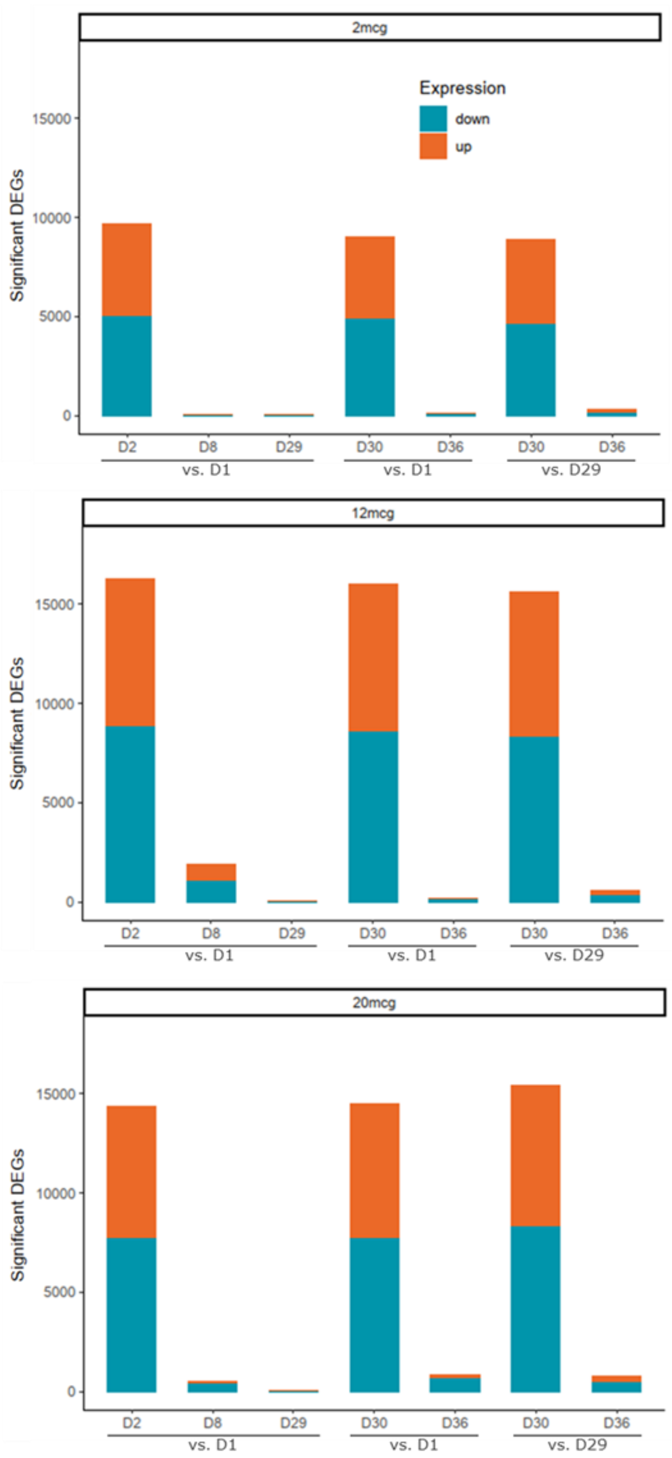

Figure S2. Significantly Differentially Expressed Genes Between Different Time Points per CVnCoV Dose Group. Numbers of significantly differentially expressed genes (DEGs) between different Days (D) per dose group (from top to bottom 2, 12 and 20 µg of CVnCoV). Significance level for adjusted p-value <0.05; downregulated DEGs in turquoise and upregulated in orange. Analysis from at least n=11 per dose group who were SARS-CoV-2 seronegative at shown timepoints. For participants who did not receive a second CVnCoV dose, Day 30 and 36 timepoint values were excluded from analysis.

Sensitivity: Official Use

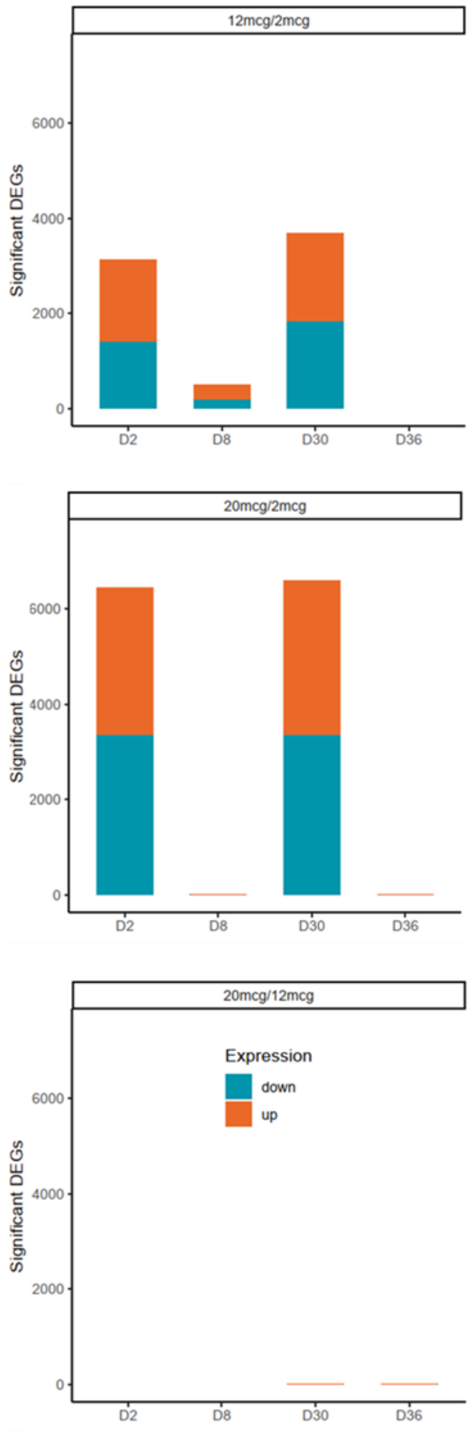

Figure S3. Significantly Differentially Expressed Genes on Different Days Between CVnCoV Dose Group. Numbers of significantly differentially expressed genes (DEGs) between different CVnCoV dose groups (from top to bottom: 12 vs. 2 µg, 20 vs. 2 µg and 20 vs. 12 µg) on one Day (D). Significance level for adjusted p-value <0.05; downregulated DEGs in turquoise and upregulated in orange. Analysis from at least n=11 per dose group who were SARS-CoV-2 seronegative at shown timepoints. For participants who did not receive a second CVnCoV dose, Day 30 and 36 timepoint values were excluded from analysis.

Sensitivity: Official Use

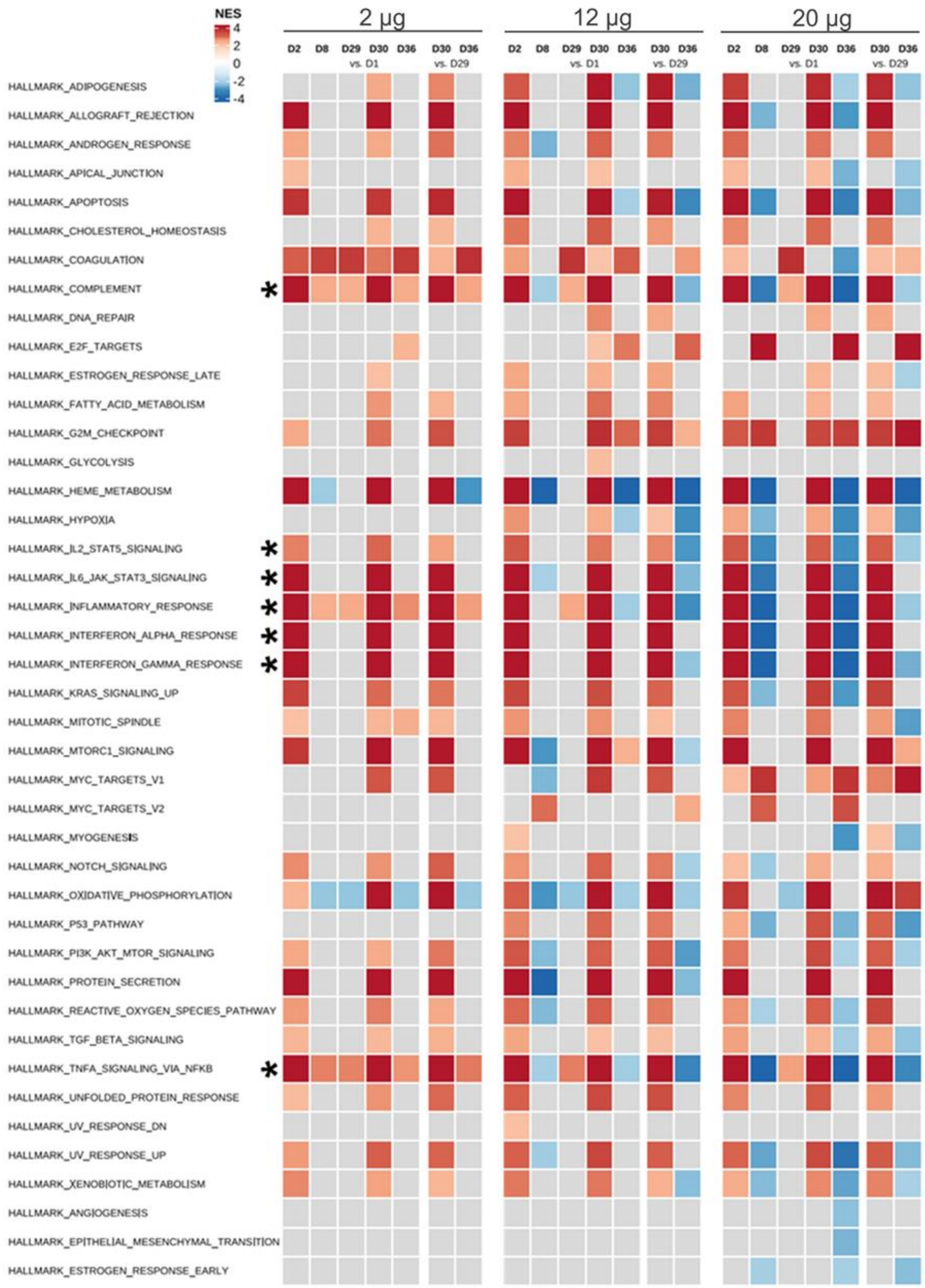

Figure S4. GSEA with HALLMARK Gene Sets After CVnCoV Vaccination. Gene set enrichment analysis (GSEA) from significantly differentially expressed genes (DEGs) summarized by HALLMARK gene sets. Analysis from at least n=11 participants per 2, 12 or 20 µg CVnCoV dose group who were SARS-CoV-2 seronegative at shown timepoints (CV-NCOV-001). For participants who did not receive a second CVnCoV dose, Day 30 and 36 timepoint values were excluded from analysis. Normalized enrichment score (NES) in grey when not enriched in GSEA or with non-significant false discovery rate (FDR > 0.01); colored NES indicates significance with FDR <0.01, red with positive score indicates upregulation and blue (negative score) downregulation of listed gene sets between compared groups. * indicates gene sets related to inflammatory processes.

Sensitivity: Official Use

D, Day; IL, interleukin; KRAS, 15kirsten rat sarcoma viral oncogene homolog; MYC, myelocytomatosis oncogene; n, number of participants; NFKB, nuclear factor kappa B; TGF, tumor growth factor; TNFA, tumor necrosis factor alpha.

Sensitivity: Official Use

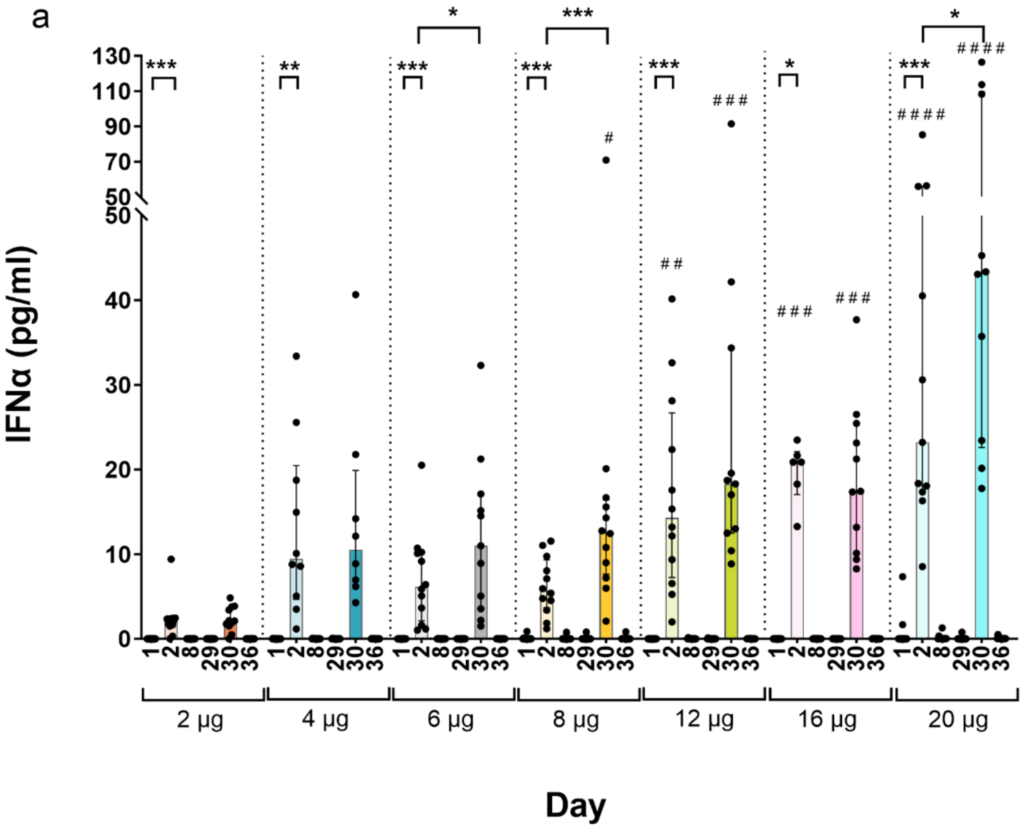

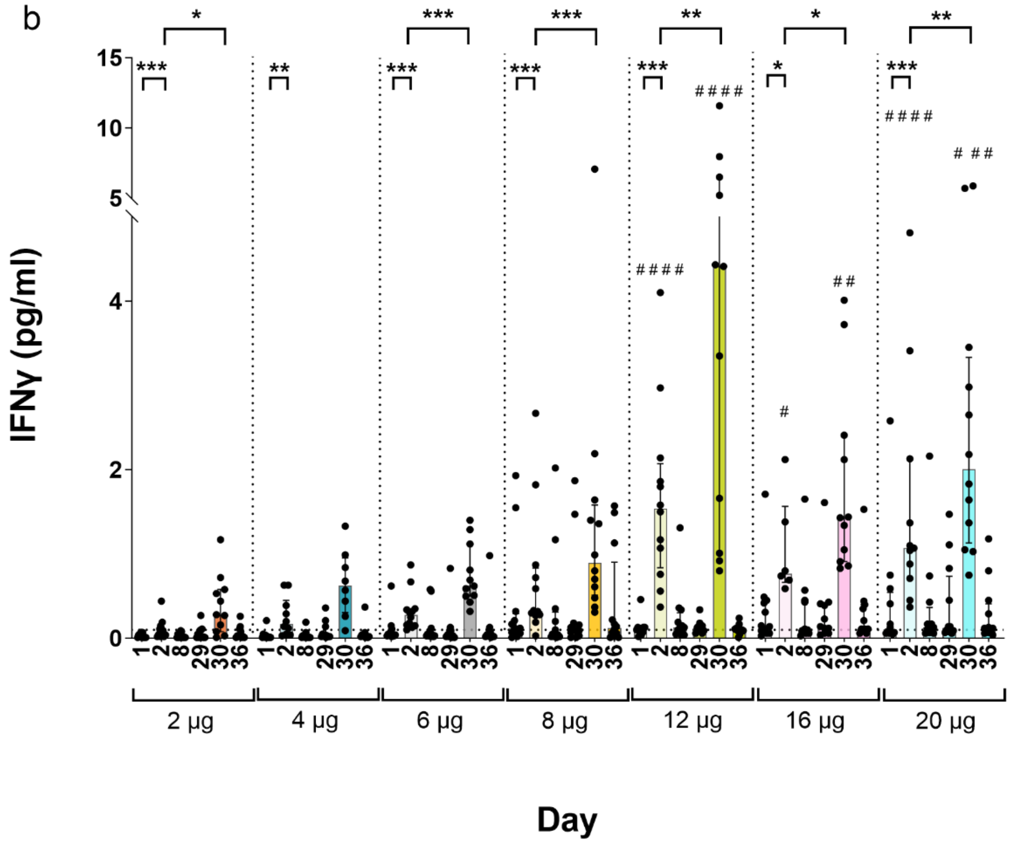

Sensitivity: Official Use

Figure S5. Serum Interferon Concentration by Dose and Trial Visit. IFNα (a) and IFNγ (b) serum concentrations on Days 1, 2, 8, 29, 30 and 36 in SARS-CoV-2 seronegative participants vaccinated with two doses of 2, 4, 6, 8, 12, 16 or 20 μg of CVnCoV (CV-NCOV-001). If participants did not receive a second CVnCoV dose, Day 30 and 36 values were excluded from analysis. Bars show median values with interquartile range. For IFNγ (b), dotted line indicates the lower level of quantification (LLOQ). Values below LLOQ were reported as extrapolated value. Values below detection limit were set to the lowest extrapolated value reported for respective cytokine. Statistical comparisons in one dose group (indicated as *) were analyzed using a two-tailed Wilcoxon signed-rank test and for Day 2 and Day 30 timepoints between dose groups using Kruskal-Wallis ANOVA with Dunn's multiple comparisons testing with 2 μg as the reference (indicated as #). Significance is defined as: */# p≤0.05, **/# # p≤0.01, ***/# # # p≤0.001 or # # # # p≤0.0001.

IFNα, interferon alpha; IFNγ, interferon gamma.

Sensitivity: Official Use

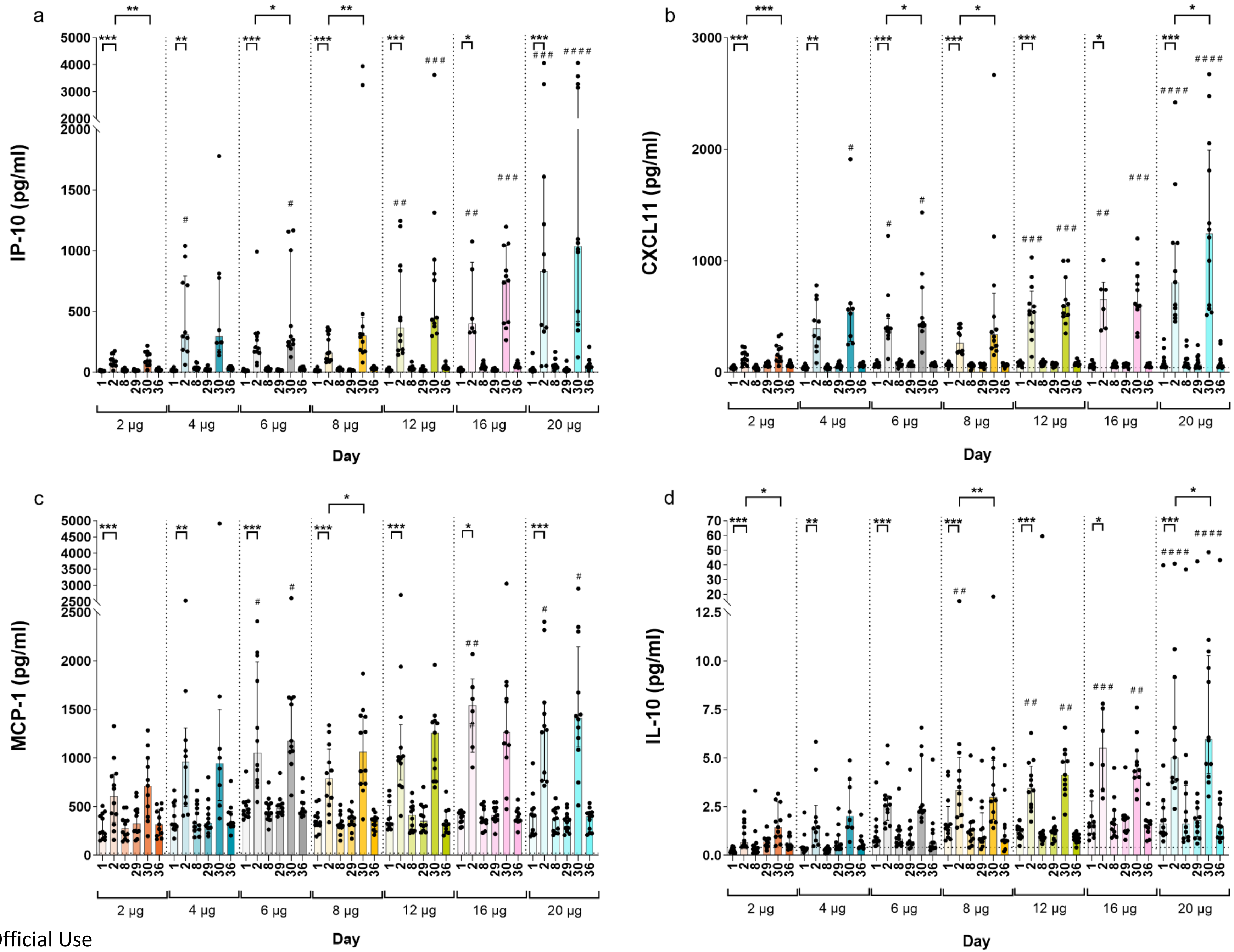

Sensitivity: Official Use

Figure S6. Induction of Serum Cytokines Post CVnCoV Vaccination. IP-10 (a), CXCL11 (b), MCP-1 (c) and IL-10 (d) serum concentrations on Days 1, 2, 8, 29, 30 and 36 in SARS-CoV-2 seronegative participants vaccinated with two CVnCoV doses of 2, 4, 6, 8, 12, 16 or 20 µg (CV-NCOV-001). If participants did not receive a second CVnCoV dose, Day 30 and 36 values were excluded from analysis. Bars show median values with interquartile range, dotted line indicates the lower level of quantification (LLOQ). Values below LLOQ were reported as extrapolated value. Values below detection limit were set to the lowest extrapolated value reported for respective cytokine. Statistical comparisons in one dose group (indicated as *) were analyzed using a two-tailed Wilcoxon signed-rank test and for Day 2 and Day 30 timepoints between dose groups using Kruskal-Wallis ANOVA with Dunn's multiple comparisons testing with 2 µg as the reference (indicated as #). Significance is defined as: */# $p \leq 0.05$, **/# # $p \leq 0.01$, ***/# # # $p \leq 0.001$ or # # # # $p \leq 0.0001$.

CXCL, chemokine (C-X-C motif) ligand 1; IL-10, interleukin 10; IP-10, interferon-gamma inducible protein 10kDa; MCP-1, membrane cofactor protein.

Sensitivity: Official Use

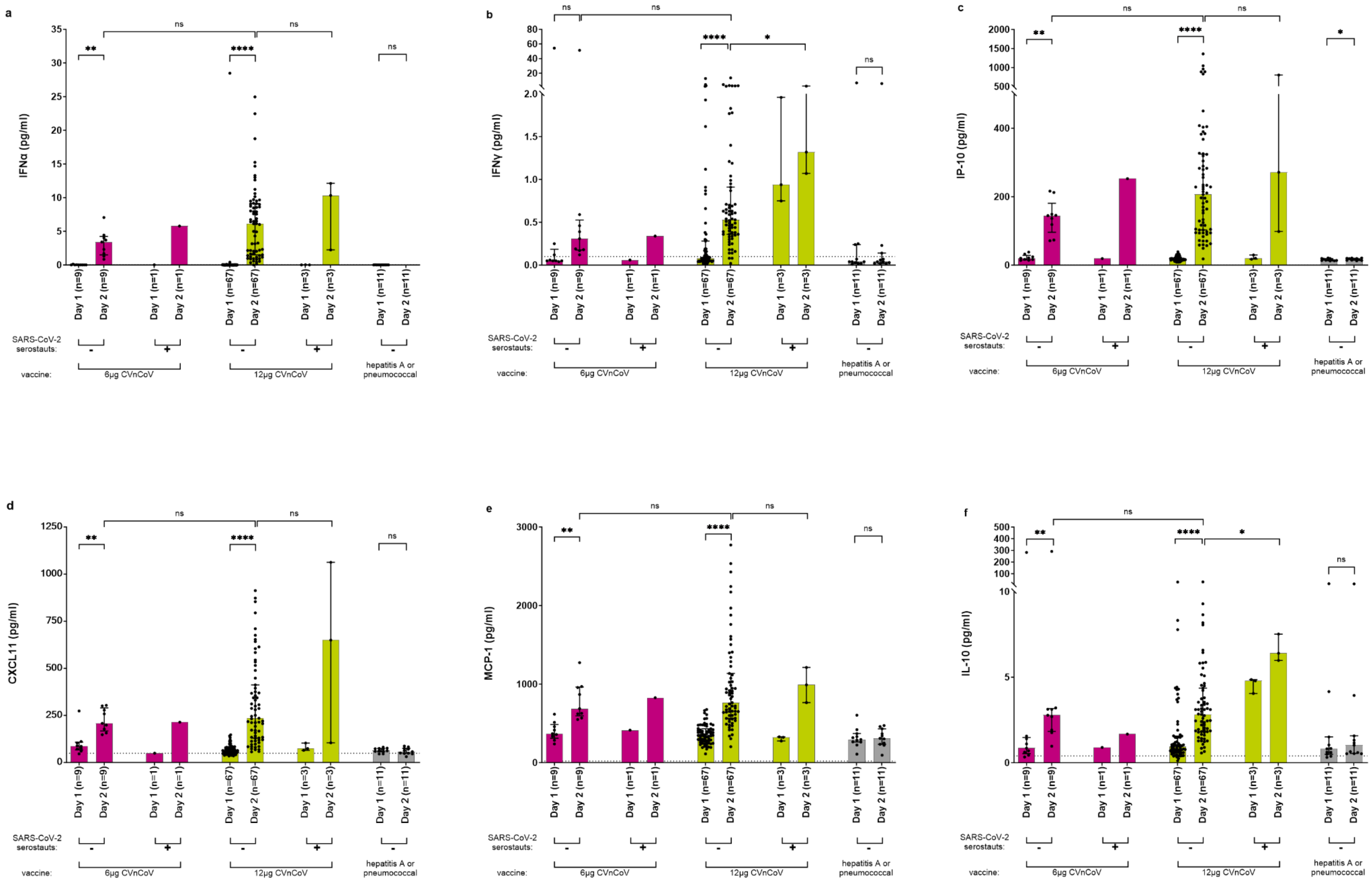

Sensitivity: Official Use

Figure S7. Serum Cytokine Concentrations Post CVnCoV Vaccination By SARS-CoV-2 Serostatus. CV-NCOV-002 cytokine serum concentrations on Day 1 and Day 2 in participants vaccinated with 6 or 12 µg of CVnCoV stratified by SARS-CoV-2 seronegative or seropositive serostatus and Active Control participants who received licensed hepatitis A or pneumococcal vaccine. Bars show median with interquartile range, dotted line indicates the lower level of quantification (LLOQ). Values below LLOQ were reported as extrapolated value. If no value was detected (below limit), the lowest extrapolated value for respective cytokine was used. Groups were compared using either a two-tailed Wilcoxon signed-rank test (pairwise comparison between Day 1 and 2 values in the same group) or by Mann-Whitney test (unpaired comparison between Day 2 values of distinct groups). Statistical significance was defined as: * $p \leq 0.05$, ** $p \leq 0.01$ or **** $p \leq 0.0001$; not significant (ns) $p > 0.05$.

CXCL, chemokine (C-X-C motif) ligand 1; IFNα, interferon alpha; IFNγ, interferon gamma; IL, interleukin; IP-10, interferon-gamma inducible protein 10kDa; MCP-1, membrane cofactor protein 1.

Sensitivity: Official Use

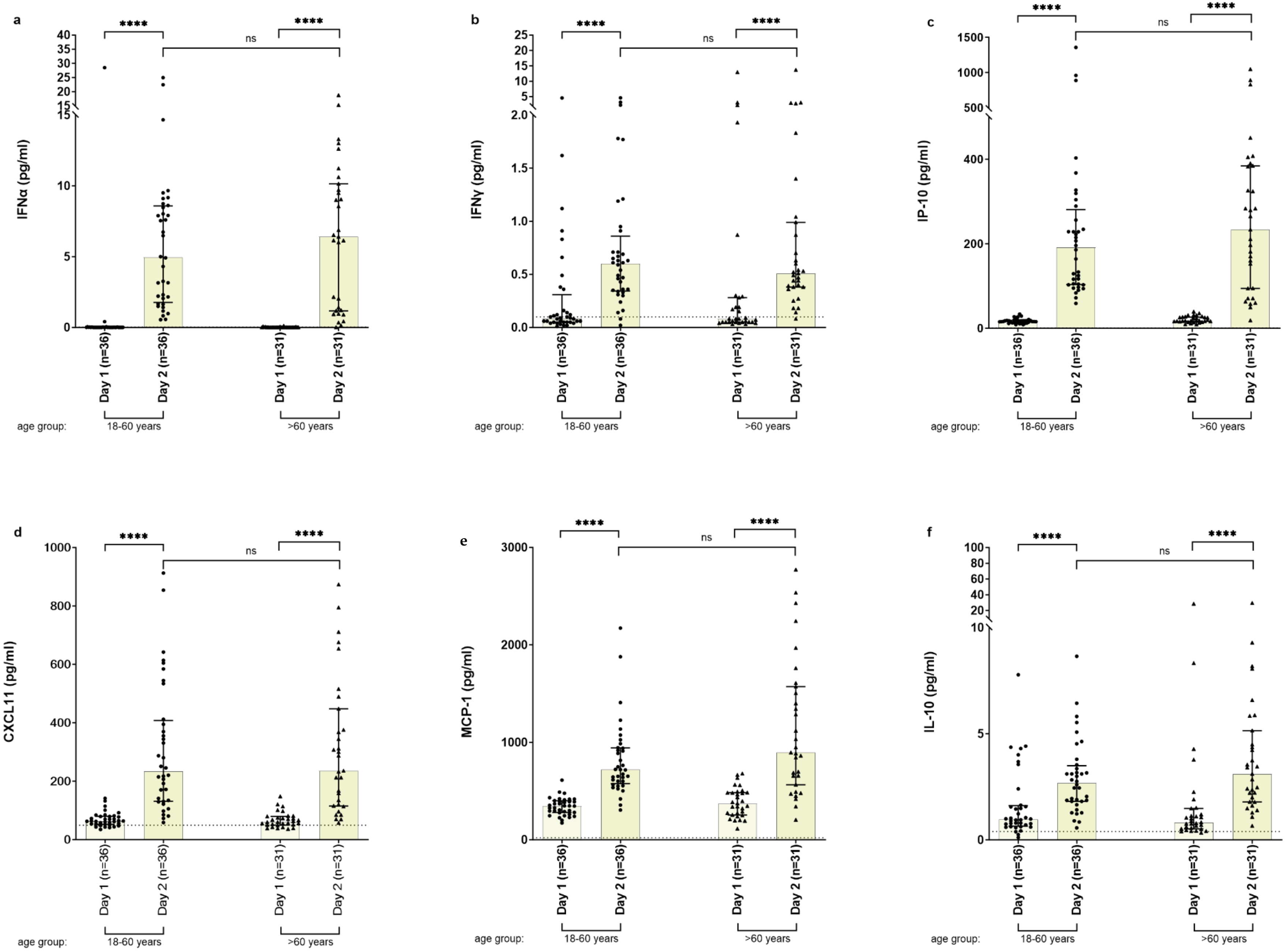

Sensitivity: Official Use

Figure S8. Serum Cytokine Concentrations by Trial Visit and Age Group. IFNα (a), IFNγ (b), IP-10 (c), CXCL11 (d), MCP-1 (e) and IL-10 (f) serum concentrations on Day 1 and Day 2 in SARS-CoV-2 seronegative participants from CV-NCOV-002 trial. Participants administrated with a 12 µg CVnCoV dose were stratified by younger (18–60 years of age) and older (over 60 years of age) groups. Bars show median values with interquartile range. Dotted lines indicate lower level of quantification (LLOQ). Values below LLOQ were reported as extrapolated value. If no value was detected (below limit), the lowest extrapolated value for respective cytokine was used. Groups were statistically compared using either a two-tailed Wilcoxon signed-rank test (pairwise comparison between Day 1 and 2 values in the same age group) or by Mann-Whitney test (unpaired comparison between Day 2 values of distinct age groups). Statistical significance was defined as: **** p≤0.0001; not significant (ns) if p>0.05.

CXCL, chemokine (C-X-C motif) ligand 1; IFNα, interferon alpha; IFNγ, interferon gamma; IL-10, interleukin 10; IP-10, interferon-gamma inducible protein 10kDa; MCP-1, membrane cofactor protein 1; n, number of participants.

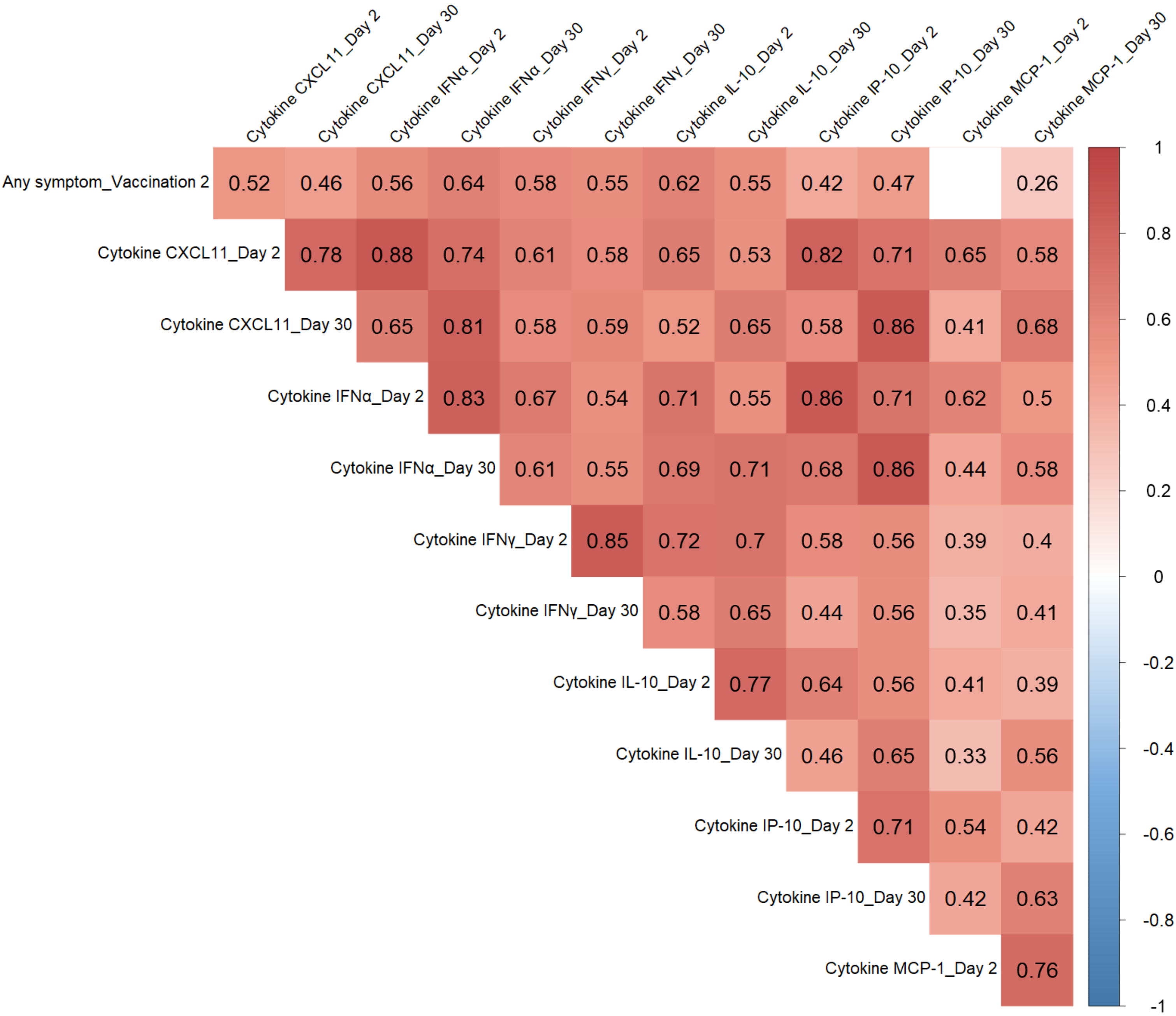

Figure S9. Correlation of Serum Cytokine Levels After First and Second Doses of CVnCoV Vaccine. Spearman correlation analysis from n=65 from a pool of CV-NCOV-001 participants who received both two doses of CVnCoV (2,4,6,8,12,16 or 20 µg) and who were SARS-CoV-2 seronegative for shown time points. Fields with significant $p \leq 0.05$ are colored according to Spearman correlation coefficient. Reactogenicity was calculated per participant as maximum severity grade (0=absent, 1=mild, 2=moderate, 3=severe) of vaccine-related solicited adverse events (any local or systemic sign/symptom) after both doses. Analysis done with serum cytokine concentrations on indicated days.

CXCL, chemokine (C-X-C motif) ligand 1; IFNα, interferon alpha; IFNg, interferon gamma; IL-10, interleukin 10; IP-10, interferon-gamma inducible protein 10kDa; MCP-1, membrane cofactor protein 1; n, number of participants.

Sensitivity: Official Use

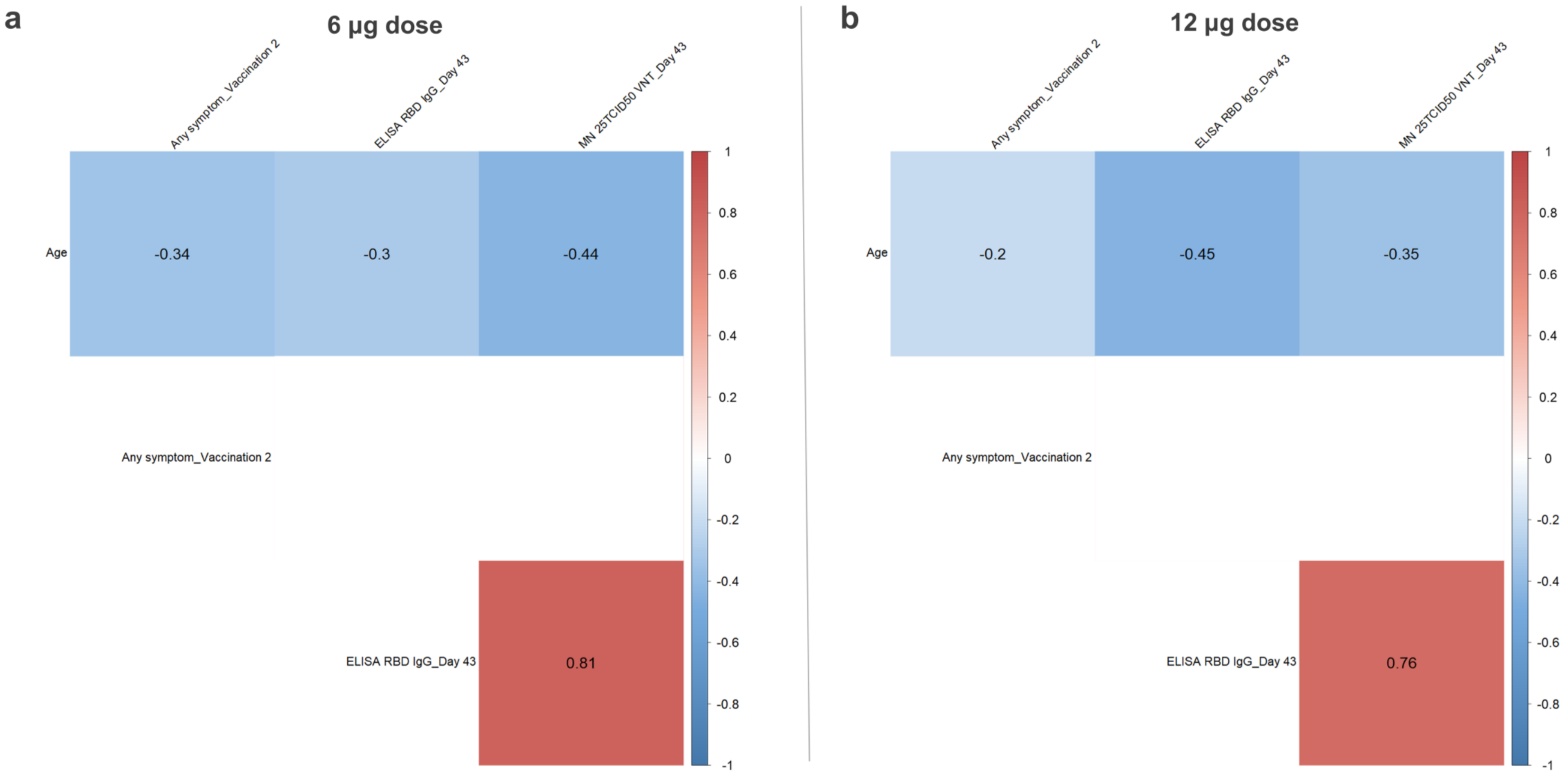

Sensitivity: Official Use

Figure S10. Correlation of CVnCoV Reactogenicity and Humoral Immunity with Participant's Age. Spearman correlation analysis from n=58 (a, 6 µg) and n=344 (b, 12 µg) pairs from a pool of CV-NCOV-001 and -002 participants who received both CVnCoV doses and who were SARS-CoV-2 seronegative for shown time points. Fields with significant $p \leq 0.05$ are colored according to Spearman correlation coefficient. Reactogenicity was calculated per participant as maximum severity grade (0=absent, 1=mild, 2=moderate, 3=severe) of vaccine-related solicited adverse events (any local or systemic sign/symptom) after both doses. Analysis performed using VNT and RBD IgG assay titers on indicated days and participants' age in years.

ELISA, enzyme-linked immunosorbent assay; IgG, immunoglobulin G; n, number of participants; RBD, receptor binding domain; VNT, virus neutralizing antibody.

Sensitivity: Official Use

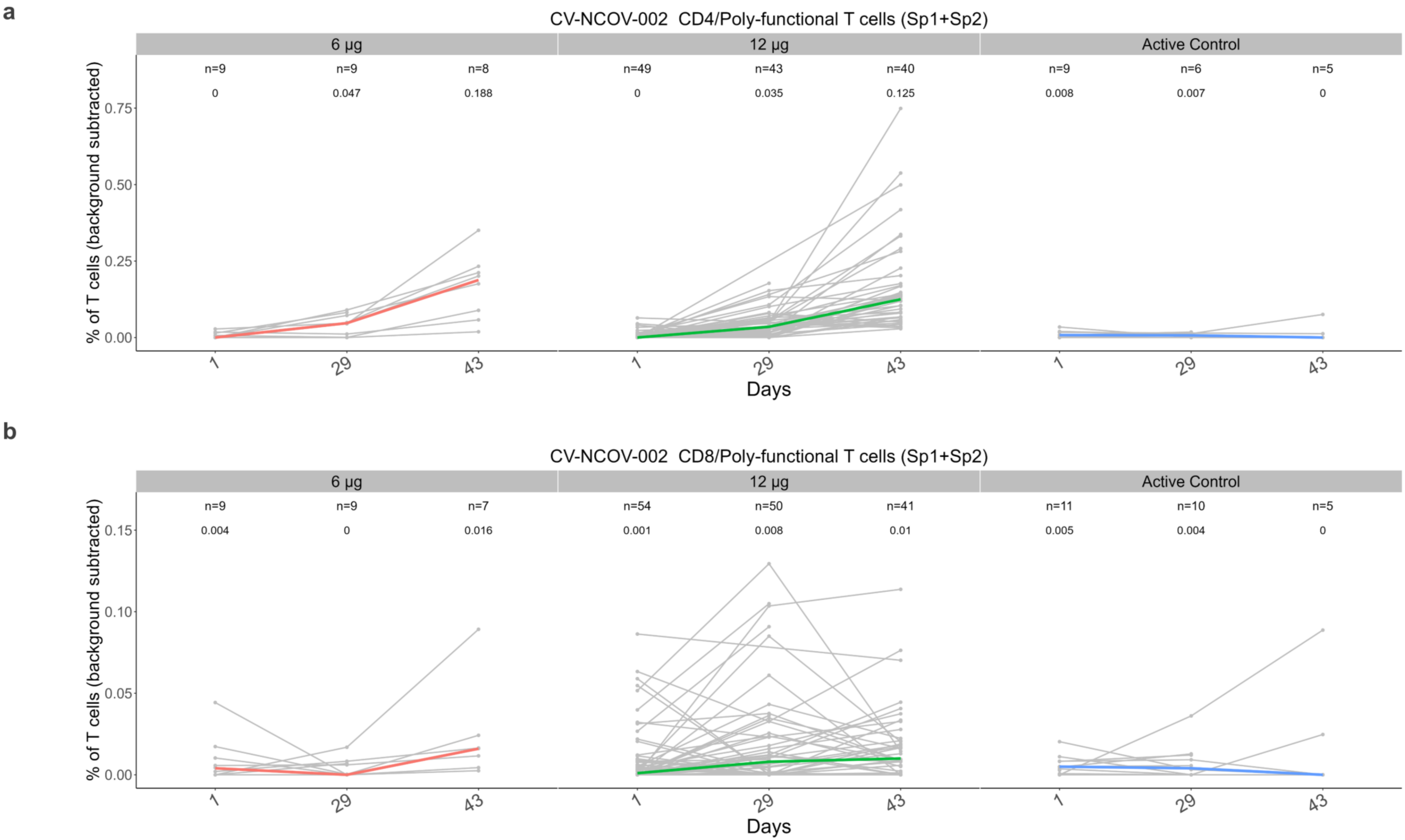

Sensitivity: Official Use

Figure S11. Ex Vivo ICS Analysis of Spike-Specific $CD4^+$ and $CD8^+$ T Cell Responses Post Vaccination in CV-NCOV-002 clinical trial. SARS-CoV-2-spike-specific $CD4^+$ and $CD8^+$ T cells were measured by intracellular cytokine staining after stimulation with individual spike pool 1 (Sp1) and spike pool 2 (Sp2). The total T cell frequencies were plotted for Day 1, Day 29 and Day 43 after participants received CVnCoV (6 or 12 μg) or an active control vaccine. Data from participants who were seronegative at baseline are shown. Values from individual participants are represented by grey symbols connected by grey lines, whilst colored lines display group medians. Values above data points indicate median of $CD4^+$ (a) and $CD8^+$ (b) T cells frequencies per time point.

ICS, intracellular cytokine staining; n, number of participants.

Sensitivity: Official Use

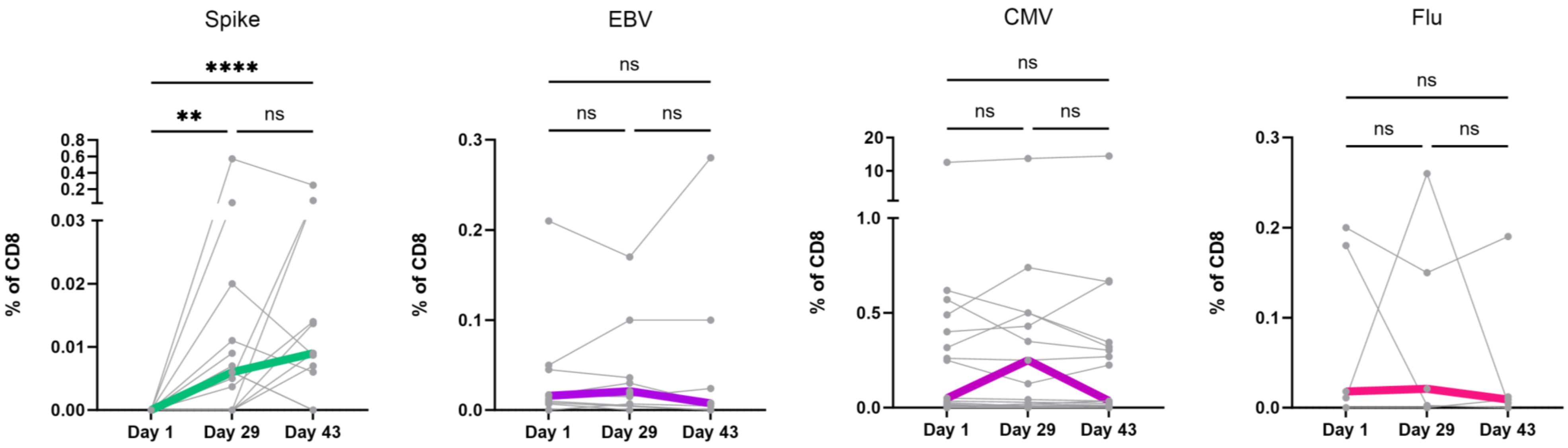

Figure S12. Frequencies of Antigen-Specific CD8+ T cells in Longitudinal Samples from CVnCoV Vaccinated Trial Participants. Virus specific $CD8^+$ T cell responses were assessed using pooled data from CV-NCOV-002 participants vaccinated with different dose levels of CVnCoV (6 and 12 µg). HLA class I tetramer peptide antigens derived from CMV, EBV, influenza virus (Flu) or SARS-CoV-2 spike protein were used to stimulate antigen-specific $CD8^+$ T cell responses. The percentage of CD8+ T cells for individual participants are represented by grey symbols connected by grey lines, whilst colored lines display group medians. Statistical analysis was performed using Kruskal Wallis test followed by Dunn's test for multiple comparisons. Statistical significance defined as ** $p \leq 0.01$, *** $p \leq 0.001$ or not significant (ns) if $p > 0.05$.

CMV, cytomegalovirus; EBV, Epstein-Barr virus; Flu, influenza; ns, not significant.

Sensitivity: Official Use

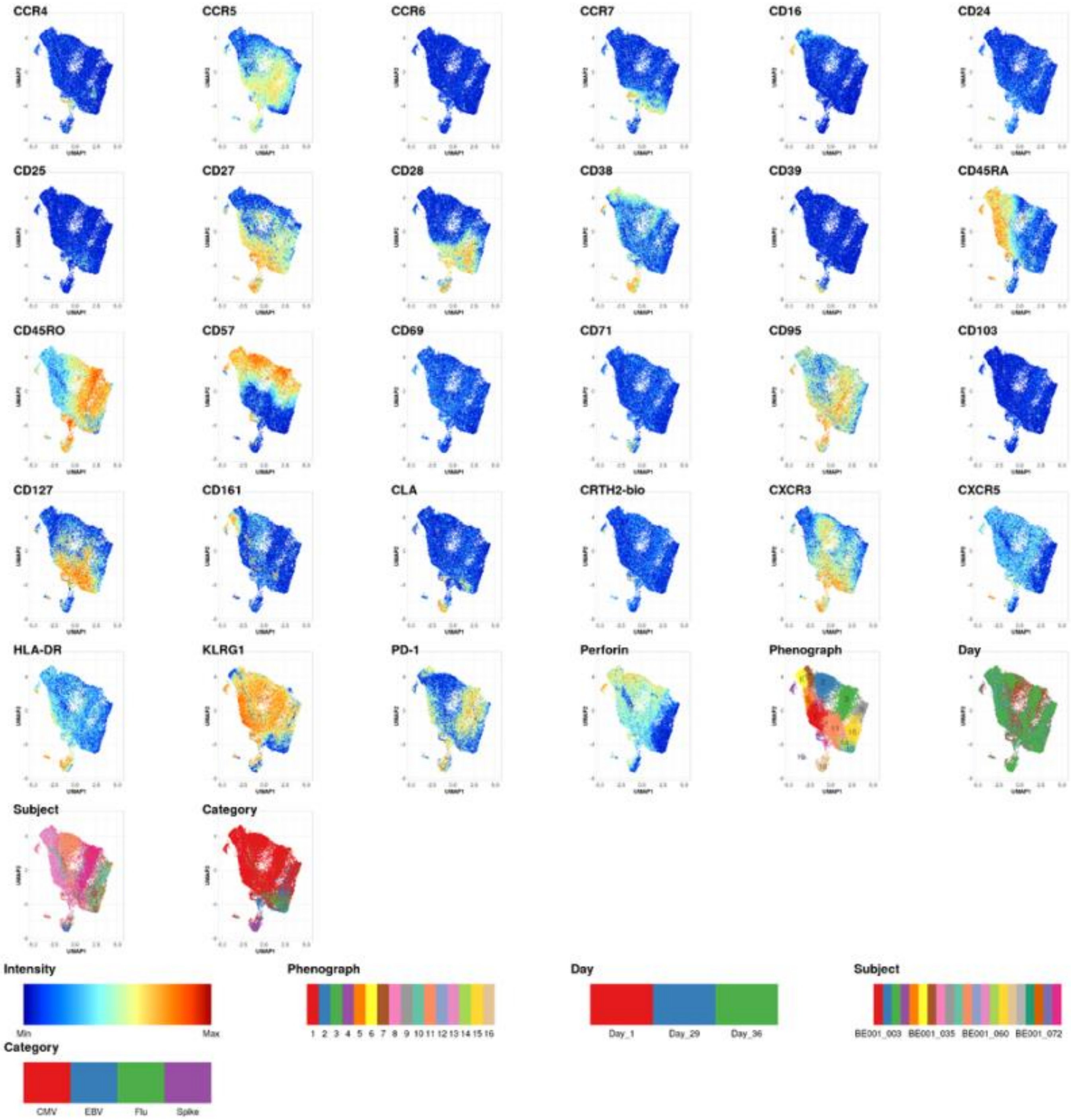

Figure S13. Phenotypes of Antigen-Specific CD8+ T cells From CVnCoV Vaccinated Individuals. UMAP plots display the expression intensity of phenotypical markers in CD8+ T cells specific to HLA class I tetramer peptide antigens from subjects analyzed in CV-NCOV-001. Category shows the HLA class I tetramer peptide antigens derived from CMV, EBV, or Influenza virus (Flu) controls or from SARS-CoV-2 Spike protein. Phenograph overlay created by unsupervised clustering algorithm.

CMV, cytomegalovirus; EBV, Epstein-Barr virus; Flu, influenza.

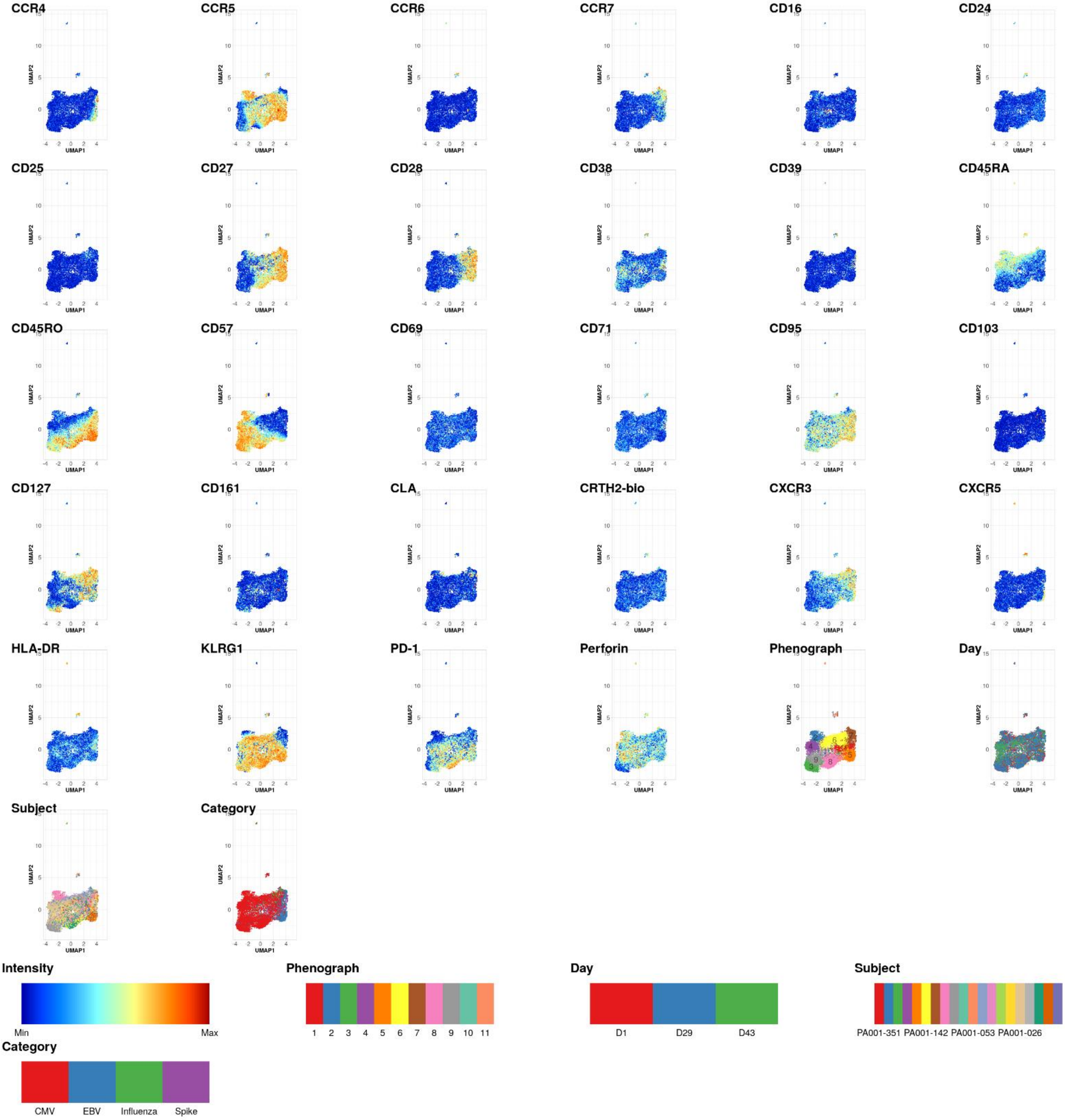

Figure S14. Phenotypes of Antigen-Specific CD8+ T cells From CVnCoV Vaccinated Individuals. UMAP plots display the expression intensity of phenotypical markers in CD8+ T cells specific to HLA class I tetramer peptide antigens from subjects analyzed in CV-NCOV-002. Category shows the HLA class I tetramer peptide antigens derived from CMV, EBV, or Influenza virus controls or from SARS-CoV-2 Spike protein. Phenograph overlay created by unsupervised clustering algorithm.

CMV, cytomegalovirus; EBV, Epstein-Barr virus.

Sensitivity: Official Use

References

Disclaimer/Publisher's Note: The statements, opinions and data contained in all publications are solely those of the individual author(s) and contributor(s) and not of MDPI and/or the editor(s). MDPI and/or the editor(s) disclaim responsibility for any injury to people or property resulting from any ideas, methods, instructions or products referred to in the content.

1. Love, M.I.; Huber, W.; Anders, S. Moderated estimation of fold change and dispersion for rna-seq data with deseq2. *Genome Biology* **2014**, *15*, 550, doi:10.1186/s13059-014-0550-8.
2. Team, R.C. R: A language and environment for statistical computing. R foundation for statistical computing, vienna, austria. Available online: https://www.R-project.org/. (accessed on 1 April 2024)
3. Benjamini, Y.; Hochberg, Y. Controlling the false discovery rate: A practical and powerful approach to multiple testing. *Journal of the Royal Statistical Society: Series B (Methodological)* **1995**, *57*, 289-300, doi:10.1111/j.2517-6161.1995.tb02031.x.
4. Subramanian, A.; Tamayo, P.; Mootha, V.K.; Mukherjee, S.; Ebert, B.L.; Gillette, M.A.; Paulovich, A.; Pomeroy, S.L.; Golub, T.R.; Lander, E.S.; et al. Gene set enrichment analysis: A knowledge-based approach for interpreting genome-wide expression profiles. *Proc Natl Acad Sci U S A* **2005**, *102*, 15545-15550, doi:10.1073/pnas.0506580102.
5. Mootha, V.K.; Lindgren, C.M.; Eriksson, K.-F.; Subramanian, A.; Sihag, S.; Lehar, J.; Puigserver, P.; Carlsson, E.; Ridderstråle, M.; Laurila, E.; et al. Pgc-1α-responsive genes involved in oxidative phosphorylation are coordinately downregulated in human diabetes. *Nature Genetics* **2003**, *34*, 267-273, doi:10.1038/ng1180.
6. Li, S.; Rouphael, N.; Duraisingham, S.; Romero-Steiner, S.; Presnell, S.; Davis, C.; Schmidt, D.S.; Johnson, S.E.; Milton, A.; Rajam, G.; et al. Molecular signatures of antibody responses derived from a systems biology study of five human vaccines. *Nature Immunology* **2014**, *15*, 195-204, doi:10.1038/ni.2789.
7. Liberzon, A.; Birger, C.; Thorvaldsdóttir, H.; Ghandi, M.; Mesirov, J.P.; Tamayo, P. The molecular signatures database (msigdb) hallmark gene set collection. *Cell Syst* **2015**, *1*, 417-425, doi:10.1016/j.cels.2015.12.004.
8. Reimand, J.; Isserlin, R.; Voisin, V.; Kucera, M.; Tannus-Lopes, C.; Rostamianfar, A.; Wadi, L.; Meyer, M.; Wong, J.; Xu, C.; et al. Pathway enrichment analysis and visualization of omics data using g:Profiler, gsea, cytoscape and enrichmentmap. *Nature Protocols* **2019**, *14*, 482-517, doi:10.1038/s41596-018-0103-9.
9. Gu, Z.; Eils, R.; Schlesner, M. Complex heatmaps reveal patterns and correlations in multidimensional genomic data. *Bioinformatics* **2016**, *32*, 2847-2849, doi:10.1093/bioinformatics/btw313.
10. Wickham, H. *Ggplot2. Elegant graphics for data analysis*; Spinger: Berlin/Heidelberg, Germany 2009.

Sensitivity: Official Use
